# *Angelica sinensis* polysaccharide nanoparticles can improve myocardial ischemia-reperfusion injury by inhibiting ferritinophagy via the ATF6/NCOA4 pathway

**DOI:** 10.1186/s12967-026-07752-8

**Published:** 2026-02-26

**Authors:** Cheng Chen, Jing Zhao, Maomao Zhao, Shuwen Hu, Pei Wang, Peng Lei, Yongxiang Wang, Yu Peng, Ming Bai, Xiaowei Niu, Zheng Zhang

**Affiliations:** 1 The First School of Clinical Medicine, Lanzhou University, Lanzhou, Gansu 730000 China; 2 Department of Cardiology, The First Hospital of Lanzhou University, No. 1, Donggang West Road, Lanzhou, Gansu 730000 China; 3 Gansu Key Laboratory of Cardiovascular Diseases, The First Hospital of Lanzhou University, Lanzhou, Gansu 730000 China; 4 Gansu Clinical Medical Research Center for Cardiovascular Diseases, The First Hospital of Lanzhou, Lanzhou, Gansu 730000 China

**Keywords:** Myocardial ischemia-reperfusion injury, *Angelica sinensis* polysaccharides, ROS, ATF6, Ferroptosis

## Abstract

**Background:**

Ferroptosis aggravates myocardial ischemia-reperfusion injury (MI/RI) by disrupting iron homeostasis, accelerating lipid peroxidation, and elevating reactive oxygen species (ROS) levels. Although *Angelica sinensis* polysaccharide (ASP) has shown protective effects against MI/RI, its clinical translation remains limited due to poor bioavailability and low target specificity.

**Methods:**

To address these limitations, we developed ASP@PLGA-PEG nanoparticles using a solvent evaporation method and characterized their morphology, size distribution, and surface charge by transmission electron microscopy, dynamic light scattering, and zeta potential analysis. The protective effects of ASP@PLGA-PEG were first evaluated in HL-1 cardiomyocytes subjected to oxygen–glucose deprivation/reoxygenation (OGD/R) by assessing cell viability, mitochondrial membrane potential, ROS generation, lipid peroxidation, and antioxidant capacity. An ex vivo MI/RI model was then established using a Langendorff isolated heart perfusion system to assess hemodynamic function, infarct size, histopathology, and mitochondrial ultrastructure. In addition, an in vivo mouse MI/R model induced by LAD ligation–reperfusion was used to evaluate cardiac function, infarct size, serum injury markers, oxidative stress, and ferroptosis-/ER stress–related proteins. Finally, siRNA-mediated ATF6 knockdown was performed in HL-1 cells to determine whether the protective and anti-ferroptotic effects of ASP@PLGA-PEG are ATF6 dependent.

**Results:**

ASP@PLGA-PEGnanoparticles significantly reduced oxidative stress, improved cardiomyocyteviability, and inhibited ferroptosis in OGD/R-injured HL-1 cells. In theLangendorff model, ASP@PLGA-PEG treatment effectively decreased myocardialinfarct size, preserved cardiac hemodynamics, and alleviated structural damage.Consistently, in vivo administration of ASP@PLGA-PEG markedly improved leftventricular systolic function, reduced infarct size and serum LDH levels, preserved mitochondrial and histologicalintegrity, and restored redox homeostasis in MI/R hearts. Mechanistically,ASP@PLGA-PEG nanoparticles activated ATF6 signaling and attenuated ER stress,while suppressing NCOA4-mediated ferritinophagy, thereby limiting iron overloadand lipid peroxidation to protect cardiomyocytes against ferroptosis duringMI/RI. Importantly, ATF6 knockdown largely abrogated the effects ofASP@PLGA-PEG on NCOA4/FTH1 expression, ROS production, and lipid peroxidation,indicating that these protective actions are critically ATF6 dependent.

**Conclusions:**

Thisstudy demonstrates that ASP@PLGA-PEG nanoparticles exert potentcardioprotective effects in vitro, ex vivo, and in vivo through a multi-targetmechanism involving ER stress modulation, enhancement of antioxidativedefenses, and inhibition of ferritinophagy-driven ferroptosis. These findingshighlight ASP@PLGA-PEG as a promising nanomedicine strategy for the preventionand treatment of myocardial ischemia–reperfusion injury.

**Supplementary Information:**

The online version contains supplementary material available at 10.1186/s12967-026-07752-8.

## Introduction

Acute myocardial infarction(AMI), one of the leading causes of death worldwide, occurs due to the acute occlusion of coronary arteries, leading to prolonged myocardial ischemia and subsequent cardiomyocyte death [1]. Although restoring myocardial blood flow is crucial in treating AMI, timely reperfusion within a short therapeutic window is often difficult in clinical practice [2, 3]. Furthermore, reperfusion itself can trigger ischemia/reperfusion (I/R) injury, which exacerbates myocardial damage [4, 5]. In AMI, cardiomyocyte injury involves several cell death mechanisms, including necrosis, apoptosis, and ferroptosis. Ferroptosis, a non-apoptotic form of cell death, is induced by iron-dependent lipid peroxidation and has drawn increasing attention in AMI research. The mechanism of ferroptosis in myocardial ischemia-reperfusion injury is primarily driven by dysregulated iron metabolism and lipid peroxidation [6–8].

disturbed iron metabolism leads to iron overload in cardiomyocytes, which produces large amounts of reactive oxygen species (ROS) via the Fenton reaction, worsening oxidative stress [9]. During reperfusion, depletion of glutathione (GSH) and inactivation of glutathione peroxidase 4 (GPX4) impair the cell’s ability to clear lipid peroxides, thereby triggering lipid peroxidation and membrane disruption [10, 11]. Mitochondrial dysfunction also contributes to further ROS accumulation and damage [12, 13]. Overall, ferroptosis exacerbates ischemia-reperfusion injury by promoting lipid peroxidation, iron overload, and ROS accumulation, leading to more extensive cardiomyocyte death. Alongside ferroptosis, necrosis and apoptosis also play significant roles in AMI pathology.

*Angelica sinensis* Polysaccharide (ASP), extracted from *Angelica sinensis*, are known for their wide range of biological activities, including antioxidant, anti-inflammatory, immune-regulating, and even anti-apoptotic effects [14–19]. These properties have been demonstrated to provide significant protection to the cardiovascular system.Ai et al. [20]. demonstrated that ASP can mitigate cerebral ischemia/reperfusion injury in rabbits through antioxidant mechanisms. Similarly, Lei et al. [21]. reported that ASP protects PC12 neuronal cells from H₂O₂-induced cytotoxicity by reducing apoptosis, lowering intracellular ROS levels, and enhancing mitochondrial membrane potential in cells treated with H₂O₂. Additionally, Niu et al. [18]. found that ASP alleviates endoplasmic reticulum stress and oxidative stress by activating the ATF6(Activating Transcription Factor 6) pathway, thereby protecting H9c2 cells from H₂O₂-induced injury.Studies suggest that ASP can reduce oxidative stress and inhibit inflammation, two key drivers of myocardial damage during ischemia-reperfusion injury. Additionally, ASP has shown potential in improving endothelial function, enhancing microcirculation, and promoting angiogenesis [22, 23]. all of which contribute to improving cardiac function after AMI.However, despite its cardioprotective effects, ASP faces several challenges. Its short half-life, poor bioavailability, and lack of tissue-specific targeting limit its efficacy [24–26]. To overcome these

limitations, We designed ASP@PLGA-PEG nanoparticles to evaluate the efficacy of ASP@PLGA-PEG on myocardial ischemia/reperfusion injury(MI/RI) and its mechanism in depth by experiment.

## Materials and methods

### Preparation and characterization of ASP@PLGA-PEG

ASP@PLGA-PEG nanoparticles were prepared using a water-in-oil-in-water (W1/O/W2) double emulsion solvent-evaporation method optimized for hydrophilic cargos. Briefly, 80 mg of PLGA-PEG

(Xarxbio, Xi’an, China) was dissolved in 2 mL dichloromethane to form the organic phase, and 10 mg ASP (Solarbio, Beijing, China) was dissolved in 200 µL deionized water to form the inner aqueous phase. The aqueous ASP solution was dispersed into the organic phase and emulsified on ice using a probe sonicator (SCIENTZ JY99-IIDN, Ningbo, China) to form a primary W1/O emulsion, which was then added into 4 mL of 1.5% (w/v) PVA solution and further sonicated to obtain the W1/O/W2 emulsion. The resulting emulsion was stirred at room temperature for 6 h to evaporate dichloromethane. Nanoparticles were collected by centrifugation, washed with water, and resuspended for subsequent characterization. Hydrodynamic diameter, PDI, and zeta potential were measured by DLS (Brookhaven

90Plus PALS, USA), and morphology was observed by TEM (FEI Tecnai G2 Spirit Bio-TWIN, USA). Encapsulation efficiency and drug loading, as well as in vitro release, were quantified by the phenol–sulfuric acid method using matrix-matched calibration. Release studies were performed using a sample-and-separate approach in deionized water at 37 °C with orbital shaking, samples were taken at predetermined time points, nanoparticles were pelleted by centrifugation, supernatants were used for ASP quantification, and fresh medium was replenished to maintain sink conditions. All experiments were conducted in triplicate.

### Ex vivo fluorescence imaging of harvested organs using the DPM system

Ex vivo fluorescence imaging was performed using the DPM system (DPM-IVFM-NIR-II, China) equipped with FITC excitation/emission channels. At predetermined timepoints after tail-vein injection of FITC-labeled ASP@PLGA-PEG nanoparticles, mice were anesthetized and euthanized, and major organs (heart, liver, spleen, lung, kidney) were harvested, briefly rinsed with cold PBS, blotted dry, and placed on a black plate with constant orientation. Organs were imaged immediately on ice using identical acquisition parameters for all samples (FITC filter set, identical exposure time, medium binning). Fluorescence intensities were quantified using the DPM analysis software by selecting regions of interest (ROI), subtracting background from the plate area, and normalizing to organ wet weight. All imaging procedures were performed in the dark to minimize photobleaching.

### Cell culture and treatment

HL-1 cardiomyocytes were maintained in DMEM medium (Gibco, Waltham, ME, USA) supplemented with 10% fetal bovine serum (FBS, Gibco). The cells were incubated in a humidified atmosphere at 37 °C with 5% CO_2_.

To establish an in vitro MI/MR model, we intervened on HL-1 cardiomyocytes for 4 hours using the hypoxia-glucose deprivation (OGD) method. Prior to the experiment, cells were cultured in a conventional incubator. After the cells grew to 80% confluence, the cells were transferred to a HeraCell VIOS 160i incubator (Thermo Fisher Scientific, Waltham, MA, USA) and hypoxia was simulated by releasing a gas mixture containing 1% O₂, 5% CO₂, and 94% N₂ (37°C). During hypoxia, HL-1 cardiomyocytes were cultured in serum-free, glucose-free DMEM medium to cut off the cellular fuel supply. In contrast, control cells were cultured under normoxic conditions with regular DMEM medium in a conventional incubator.

### Small interfering RNA transfection

According to the manufacturer’s instructions, ATF6 siRNA was transfected into HL-1 cardiomyocytes using Lipofectamine™ RNAiMAX (Thermo Fisher Scientific, USA). Briefly, siRNA and transfection reagent were mixed in serum-free Opti-MEM transfection medium and incubated for 10 min at room temperature to allow complex formation, after which the complexes were added to cells at approximately 50–60% confluence. After 6 h of transfection, the medium was replaced with fresh antibiotic-free complete medium, and the cells were maintained under standard culture conditions for subsequent experiments.

### Cell viability assay

Cell viability was assessed using the Cell Counting Kit-8 (CCK-8, Beyotime, Shanghai, China). HL-1 cardiomyocytes were seeded in 96-well plates at a density of 1 × 10^4^ cells per well and subjected to the simulated in vitro MI/RI model. Following the treatment, an appropriate volume of CCK-8 solution was added according to the kit instructions, and the cells were incubated for 2 hours.

Absorbance (OD) at 450 nm was measured using a microplate reader (Tecan, Infinite M200 PRO, Männedorf, Switzerland).

### Detection of reactive oxygen species

Reactive oxygen species (ROS) levels were measured using a ROS assay kit (Beyotime, Shanghai, China). HL-1 cardiomyocytes were cultured in confocal dishes and treated according to the experimental model. After treatment, the cells were incubated with 1 mL of DCFH-DA reagent at 37 °C for 20 minutes, following the kit instructions. After incubation, the cells were washed three times with PBS and then observed under an Olympus I×71 microscope. Fluorescence intensity was quantified using ImageJ software (version 1.51 k; Bethesda, MD, USA).

### Measurement of mitochondrial membrane potential

Mitochondrial membrane potential (MMP) was assessed using the JC-1 Mitochondrial Membrane Potential Assay Kit (Solarbio, Beijing, China). HL-1 cells were seeded into confocal dishes and treated according to the experimental model. Following treatment, the cells were incubated with 1 mL of JC-1 dye solution at 37 °C for 20 minutes. After staining, the cells were observed under an Olympus microscope. The images were analyzed using ImageJ software, and MMP was quantified by calculating the red-to-green fluorescence ratio, which reflects the ratio of JC-1 aggregates (red) to monomers (green).

### Immunofluorescence detection of 4-HNE

Immunofluorescence quantification of 4-HNE was performed on HL-1 cardiomyocytes cultured in confocal petri dishes and processed according to the experimental protocol. After fixing the cells with

4% paraformaldehyde (PFA) for 10 min, permeabilizing the cells with 0.1% Triton X-100 for 10 min, and blocking with goat serum for 30 min, the cells were incubated with 4-HNE primary antibody (Thermo Fisher Scientific, MA5-27570) at 4 °C overnight. On the following day, after washing three times with phosphate-buffered saline (PBS), HL-1 cardiomyocytes were incubated with FITC-coupled secondary antibody (Thermo Fisher Scientific) for 1 h under light protection. Then, the cells were washed again three times and restained with DAPI-containing medium to visualize the nuclei. Fluorescence imaging was performed using an Olympus I×71 microscope and quantitative analysis was performed using ImageJ software.

### Animals and treatments

C57BL/6 mice aged 6–8 weeks, weighing 22–25 g, were selected as experimental animals.The experimental animals were purchased from the Laboratory Animal Center of Lanzhou University (Lanzhou, Gansu, China).The mice were provided with unrestricted access to standard rodent chow and sterile water, and were housed in an environment with an ambient temperature of 22 ± 2 °C and a

12-hour light/dark cycle. All procedures involving animals were approved by the Animal Care Committee of the First Hospital of Lanzhou University and were performed in accordance with the National Institutes of Health Guide for the Care and Use of Laboratory Animals.

### Langendorff isolated mouse heart perfusion

Mice in the Sham and I/R groups were injected with saline via the tail vein for 3 consecutive days. Mice in the ASP group received angelica polysaccharides (20 mg/kg) via tail vein injection for 3 consecutive days. Mice in the Nps group were injected with varying doses of ASP@PLGA-PEG via the tail vein for 3 consecutive days. After the final drug injection, the mice were anesthetized with an intraperitoneal injection of 2% pentobarbital sodium. The mice were then euthanized by cervical dislocation. The hearts were rapidly excised, placed in pre-cooled Krebs-Henseleit (KH) solution(pH gases 95% O2 and 5% CO2) in mM: 4.7 KCl, 118 NaCl, 25 NaHCO3, 1.8 CaCl2, 1.4 MgSO4, 1.2

KH2PO4, 11 D-glucose, and ligated with a 22 G steel needle. Finally, the hearts were connected to the Langendorff apparatus, and retrograde perfusion was initiated.

The hearts were perfused with KH solution. A latex balloon filled with normal saline and connected to a PowerLab system (AD Instruments, Sydney, NSW, Australia) was inserted into the left ventricle through the mitral valve. After placement, the balloon was inflated to maintain a left ventricular end-diastolic pressure (LVEDP) of 5–10 mmHg. Hemodynamic parameters of the isolated hearts were continuously monitored and analyzed using the LabChart 8 data acquisition system (AD Instruments).

At the end of the stabilization period, perfusion was completely stopped for 35 minutes to induce global ischemia, followed by 60 minutes of reperfusion. During reperfusion, cardiac function was continuously monitored using LabChart software, and hemodynamic data were recorded every 10 minutes for statistical analysis.

According to the experimental protocol, healthy mice were divided into 6 experimental groups:

1. **Sham group (saline):** perfusion with KH solution for 125 min;
2. **Ischemia/reperfusion group (saline):** stabilization period for 30 min, global ischemia for 35 min, and reperfusion for 60 min;
3. **ASP group (20 mg/kg):** stabilization period for 30 min, global ischemia for 35 min, reperfusion for 60 min;
4. **NpsL group (5 mg/kg):** stabilization period for 30 min, global ischemia for 35 min, reperfusion 60 min;
5. **NpsM group (10 mg/kg):** stabilization period for 30 min, global ischemia for 35 min, reperfusion 60 min;
6. **NpsH group (20 mg/kg):** stabilization period for 30 min, global ischemia for 35 min, reperfusion 60 min;

### Surgical induction of reversible LAD ligation-induced myocardial ischemia/reperfusion in mice

Male C57BL/6 mice aged 8 weeks were randomly assigned to different groups and administered ASP (20 mg/kg) or ASP@PLGA-PEG nanoparticles (20 mg/kg) via tail vein prior to surgery. All animal procedures were performed in accordance with IACUC guidelines and were approved by the institutional ethics committee. Mice were anesthetized with isoflurane (3–4% for induction and 1.5–2.0% for maintenance), followed by orotracheal intubation and mechanical ventilation using a small animal ventilator (tidal volume 10–12 mL/kg, corresponding to ~200–300 μL per breath; respiratory rate 110–130 breaths/min). Body temperature was maintained at 37 °C throughout the procedure. After shaving and disinfecting the left anterior chest, an incision of approximately 1–1.5 cm was made at the left fourth intercostal space. The chest cavity was opened, and the pericardium was carefully dissected to expose the heart. The left anterior descending coronary artery (LAD) was identified approximately 1.5 mm below the tip of the left atrial appendage. A 8–0 silk suture was placed underneath the LAD, with a small silicone tube used as a spacer to create a reversible ligation.

Myocardial blanching and reduced wall motion distal to the ligation site indicated successful ischemia. After 30 min of ischemia, the ligature was gently released and the spacer was removed to allow reperfusion. The chest wall and skin were then closed in layers, and the mice were returned to a warmed recovery chamber following surgery.

### Echocardiographic assessment

Echocardiographic evaluation was performed using a high-resolution ultrasound imaging system (Vevo 3100, Fujifilm VisualSonics, Toronto, Canada). Mice were initially anesthetized with 2% isoflurane for induction and then transferred to the imaging platform. During data acquisition, anesthesia was maintained at 0.8–1.5% via a nose cone, and body temperature was continuously monitored and maintained. Parasternal long-axis views were obtained, and M-mode tracings were recorded at the mid-papillary muscle level. Left ventricular ejection fraction (LVEF) and left ventricular fractional shortening (LVFS) were calculated to quantitatively assess cardiac function. All echocardiographic measurements were performed by an investigator blinded to group allocation. To ensure accuracy and reproducibility, each parameter was averaged from at least three consecutive cardiac cycles. Image processing and quantitative analysis were conducted using Vevo LAB software (Fujifilm VisualSonics).

### Biochemical assessment

The content of malondialdehyde (MDA), a lipid peroxidation product, and the activities of antioxidant markers including superoxide dismutase (SOD), catalase (CAT), lactate dehydrogenase (LDH), and reduced glutathione (GSH), as well as the concentration of Fe^2 +^ in cell lysates and tissue homogenates were determined using commercial assay kits (Elabscience, Wuhan, China).

### Hematoxylin and eosin staining

Histopathological changes in myocardial tissue sections were examined by hematoxylin and eosin (H&E) staining. Following completion of the in vitro experiments on the Langendorff apparatus, the hearts were fixed in 4% paraformaldehyde for 24 hours. The tissue was then dehydrated through a graded ethanol series (70%, 90%, and 100%), cleared in xylene at 60–70 °C for 60 minutes, and embedded in paraffin. Paraffin-embedded tissues were sectioned at a thickness of 4 μm and stained using a commercial H&E staining kit (Servicebio, Wuhan, China). Two authors independently evaluated the sections under an optical microscope (Olympus IX-71) in a blinded manner. The slides were subsequently scanned using a digital slide scanner (3DHISTECH, Budapest, Hungary).

### Transmission electron microscopy

Myocardial tissue samples were prepared for transmission electron microscopy (TEM) to assess mitochondrial morphology. Briefly, tissues were initially fixed in 3% glutaraldehyde solution in 0.1 M phosphate buffer (pH7.4) at 4 °C for 2 hours to preserve cellular ultrastructure. Following primary fixation, samples were post-fixed in 1% osmium tetroxide (OsO₄; Sigma-Aldrich) in 0.1 M phosphate buffer (pH7.4) for 1hour at room temperature to enhance contrast and stabilize membrane structures.

After post-fixation, the tissues were dehydrated through a graded series of acetone solutions (30%, 50%, 70%, 90%, and 100%), 15 minutes for each step, and then infiltrated with a mixture of acetone and Epon 812 resin (SPI Supplies, West Chester, PA, USA). The tissues were subsequently embedded in pure Epon 812 resin, and polymerization was performed at 60 °C for 48 hours. Ultrathin sections (60–90 nm) were obtained using a diamond knife (Leica Microsystems, Wetzlar, Germany) on an ultramicrotome (Leica EM UC7). Sections were mounted on copper grids (200 mesh) and sequentially stained with 2% uranyl acetate (SPI Supplies) for 10 minutes and lead citrate (Electron Microscopy Sciences, Hatfield, PA, USA) for 5 minutes to increase electron density and contrast. The stained sections were examined under a transmission electron microscope (FEI Tecnai G2 Spirit Bio-TWIN, USA) at a magnification of 10,000× to capture high-resolution images of interfibrillar mitochondria. Mitochondrial morphological parameters, including size, shape, and cristae integrity, were quantified by an investigator blinded to the experimental groups.

### Western blot analysis

Cardiac tissues or cells were lysed in a mixture of RIPA buffer (Beyotime, Shanghai, China) supplemented with PMSF (Beyotime, Shanghai, China) to extract total protein. Lysates were incubated on ice for 30 minutes with periodic vortexing, and the supernatants were collected for further analysis. Protein concentration was determined using a BCA Protein Assay Kit (Elabscience, Wuhan, China) according to the manufacturer’s instructions. Equal amounts of protein were separated by 10–15%

SDS-PAGE and transferred onto PVDF membranes (Millipore, USA) in transfer buffer for 2 hours. Membranes were blocked with 5% skimmed milk or bovine serum albumin (BSA) for 2 hours at room temperature. After blocking, membranes were incubated overnight at 4 °C with the appropriate primary antibodies (see Additional File 1: Table S1 for details). After washing, the membranes were incubated with HRP-conjugated secondary antibodies (MedChemExpress, New Jersey, USA) for 2 hours at room temperature. Protein bands were visualized using an enhanced chemiluminescence (ECL) detection kit (Thermo Fisher Scientific, Waltham, MA, USA) and detected by exposure to X-ray film or captured using a digital imaging system. Band intensities were analyzed and quantified using ImageJ software (version 1.51k; NIH, Bethesda, MD, USA). Relative protein expression levels were normalized to the housekeeping protein GAPDH.

### RNA Extraction and real-time quantitative PCR analysis (qRT-PCR)

Total RNA was extracted from tissues or cells using TRIzol reagent (Invitrogen, NY, USA) according to the manufacturer’s instructions. The RNA quality and concentration were assessed using a spectrophotometer (Thermo Fisher Scientific) by measuring the absorbance ratio at 260/280 nm. For cDNA synthesis, 1 µg of total RNA was reverse-transcribed using the PrimeScript RT Reagent Kit (Takara Biotechnology, Shiga, Japan) following the manufacturer’s protocol. The reverse transcription reaction was performed in a total volume of 20 µL at 37 °C for 15 minutes, followed by inactivation at

85 °C for 5 minutes. The resulting cDNA was used as a template for quantitative real-time PCR (qRT-PCR). qRT-PCR was performed on a LightCycler 480 system (Roche Diagnostics, Burgess Hill, UK) using SYBR Green Master Mix (Roche, Basel, Switzerland). Each reaction was carried out in a final volume of 20 µL containing 10 µL of SYBR Green Master Mix, 1 µL of cDNA, 0.5 µL each of forward and reverse primers, and 8 µL of nuclease-free water. The thermal cycling conditions were as follows: an initial denaturation at 95 °C for 10 minutes, followed by 40 cycles of denaturation at 95 °C for 15 seconds, annealing at 60 °C for 30 seconds, and extension at 72 °C for 30 seconds. Relative gene expression levels were calculated using the 2^−ΔΔCt method, with GAPDH used as the internal reference gene. The primers used for the target genes are listed below:

ATF6:5’-CAGCAAAGACCATCATCATTCAG-3‘and5’-TTAGTCACACACAGTTTTCCGTTC-3‘GAPDH:5’-AGGTCGGTGTGAACGGATTTG-3‘and5’-TGTAGACCATGTAGTTGAGGTCA-3’.

### Statistical analysis

All data are presented as the mean ± standard deviation (SD). For comparisons between two independent groups, an unpaired Student’s t-test was performed. For comparisons among multiple groups, one-way analysis of variance (ANOVA) followed by Tukey’s post hoc test was conducted to determine specific pairwise differences. A P-value of less than 0.05 was considered statistically significant. All statistical analyses were performed using GraphPad Prism version 8.0 (GraphPad Software, San Diego, CA, USA).

## Results

### Characterization of ASP@PLGA-PEG

**Fig. 1:**
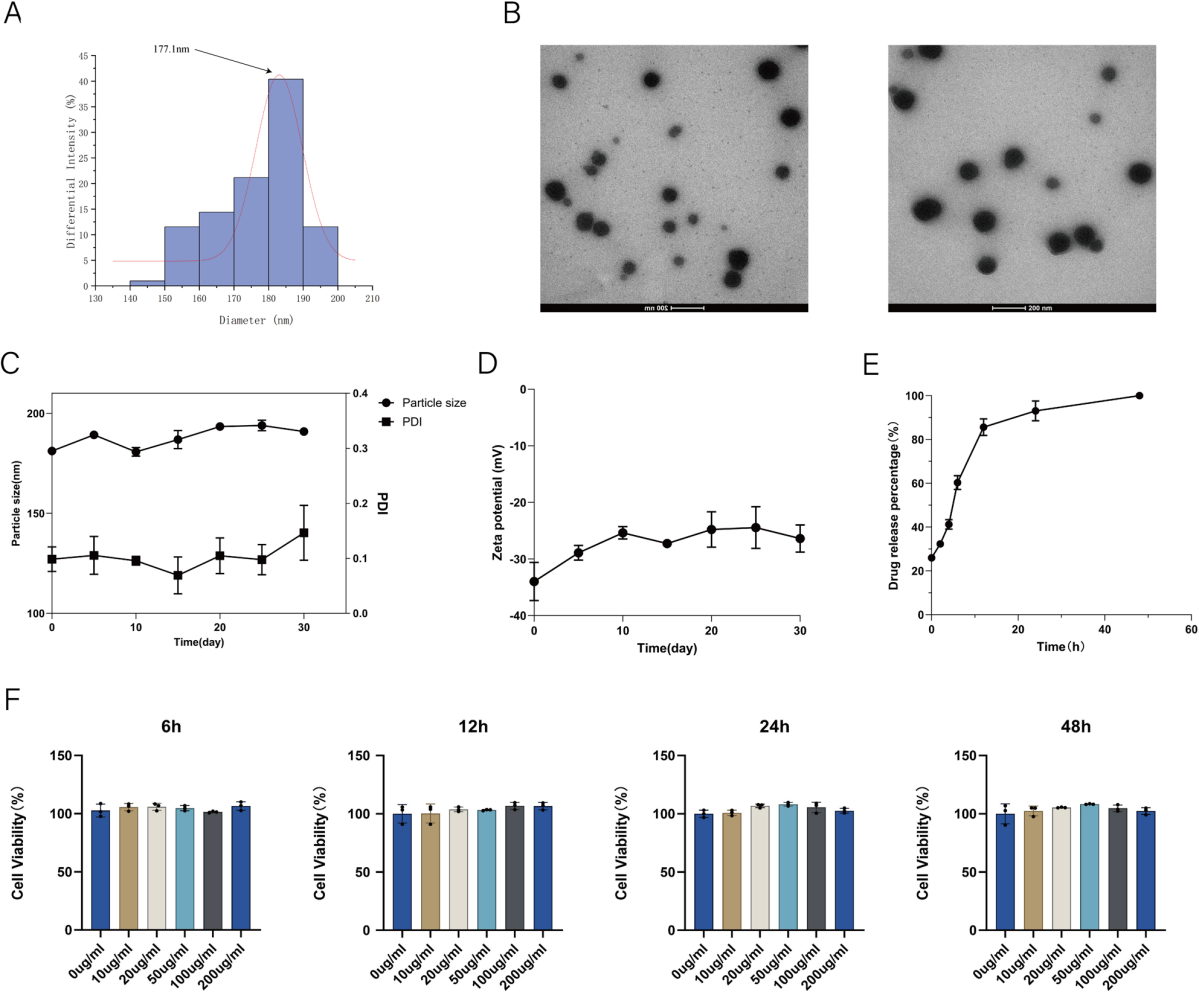
Characterization and biocompatibility evaluation of ASP@PLGA-PEG nanoparticles. (**A**) Particle size distribution of ASP@PLGA-PEG nanoparticles measured by dynamic light scattering (DLS); the average diameter was approximately 177.1 nm. (**B**) Representative transmission electron microscopy (TEM) images showing the morphology of ASP@PLGA-PEG nanoparticles; scale bars = 200 nm. (**C**) Stability profile of ASP@PLGA-PEG nanoparticles in terms of particle size and polydispersity index (PDI) over 30 days. (**D**) Zeta potential of ASP@PLGA-PEG nanoparticles measured over 30 days to assess colloidal stability. (**E**) In vitro drug release profile of ASP from ASP@PLGA-PEG nanoparticles at 37 °C. (**F**) Cell viability of HL-1 cardiomyocytes treated with various concentrations (0, 10, 20, 50, 100, and 200 μg/mL) of ASP@PLGA-PEG nanoparticles for 6, 12, 24, and 48 hours, measured by CCK-8 assay. All data are presented as mean ± SD (*n* = 3 per group)

TEM showed uniformly spherical, well-dispersed nanoparticles without aggregation (Fig. 1B).

The mean diameter by DLS was 177.1 ± 12.4 nm (Fig. 1A) and the zeta potential was −28.64 mV (Fig. 1C–D), consistent with good colloidal stability. Encapsulation efficiency and drug loading, determined by the phenol–sulfuric acid method, were 50.9% and 6.72%, respectively. Particle size and zeta potential remained stable over 30 days in water (Fig. 1C–D), likely due to electrostatic repulsion among negatively charged particles; modest destabilization thereafter may reflect PLGA-PEG degradation. In vitro release displayed an initial burst (~80% within 12 h) followed by a slower, sustained phase approaching a plateau by 48 h (Fig. 1E). ASP@PLGA-PEG showed no detectable cytotoxicity to HL-1 cardiomyocytes across 0–200 µg/mL for up to 48 h (Fig. 1F).

**Fig. 2:**
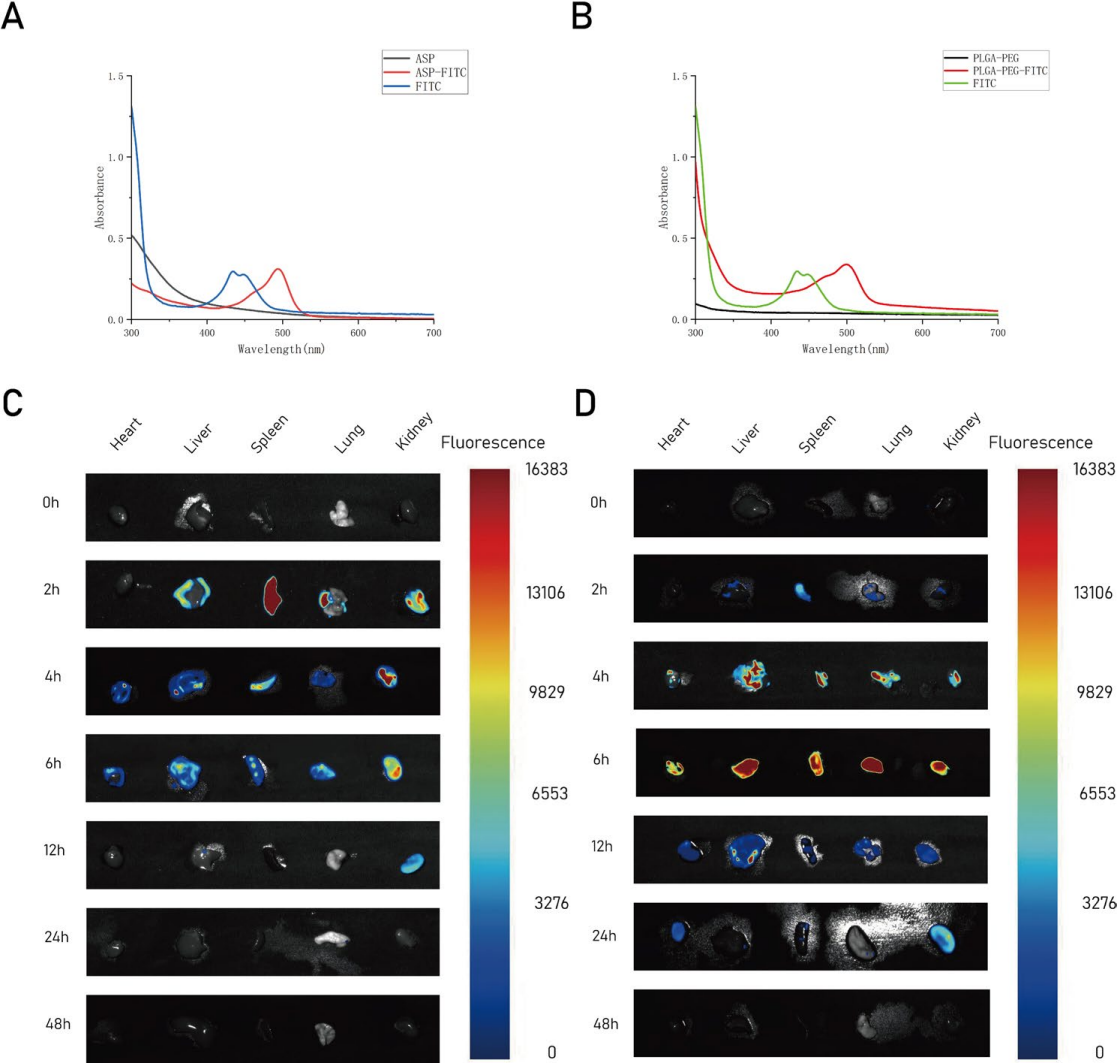
UV–vis characterization of FITC-labeled materials and ex vivo biodistribution of ASP and ASP@PLGA-PEG. (**A**) UV–vis absorption spectra of ASP, FITC, and ASP-FITC. (**B**) UV–vis absorption spectra of PLGA-PEG, FITC, and PLGA-PEG-FITC. (**C**) Ex vivo fluorescence imaging of major organs (heart, liver, spleen, lung, kidney) at different time points following intravenous administration of ASP-FITC. (**D**) Ex vivo fluorescence imaging of major organs following administration of PLGA-PEG-FITC nanoparticles. (*n* = 3 per group)

In vitro release experiments demonstrated that ASP@PLGA-PEG nanoparticles exhibited a sustained-release profile, prompting us to further investigate whether ASP@PLGA-PEG could prolong the metabolic retention time of ASP in vivo. To this end, FITC-labeled ASP and FITC-labeled ASP@PLGA-PEG nanoparticles were synthesized, and an ex vivo fluorescence imaging assay was performed to evaluate their biodistribution and metabolic behavior in mice at different time points. As shown in Fig. 2 A–B, UV–vis spectra confirmed the successful conjugation of FITC to ASP and PLGA-PEG, with both ASP-FITC and PLGA-PEG-FITC displaying characteristic FITC absorbance peaks in the 450–550 nm region.

Ex vivo organ fluorescence imaging demonstrated distinct biodistribution and clearance kinetics between free ASP and nanoparticle-encapsulated ASP (Fig. 2 C–D). For ASP-FITC, fluorescence signals were predominantly detected in the liver and spleen at early time points (2–6 h), followed by rapid attenuation toward baseline by 48 h. In contrast, PLGA-PEG-FITC nanoparticles showed markedly prolonged retention, with strong fluorescence persisting in the liver and spleen up to 12–24 h, and detectable signal still present at 48 h. These results indicate that nanoparticle encapsulation significantly extends the in vivo residence time of ASP, consistent with the sustained-release behavior observed in vitro.

### Langendorff isolated heart perfusion demonstrates that ASP@PLGA-PEG improves cardiac functional recovery after ischemia/reperfusion

**Fig. 3:**
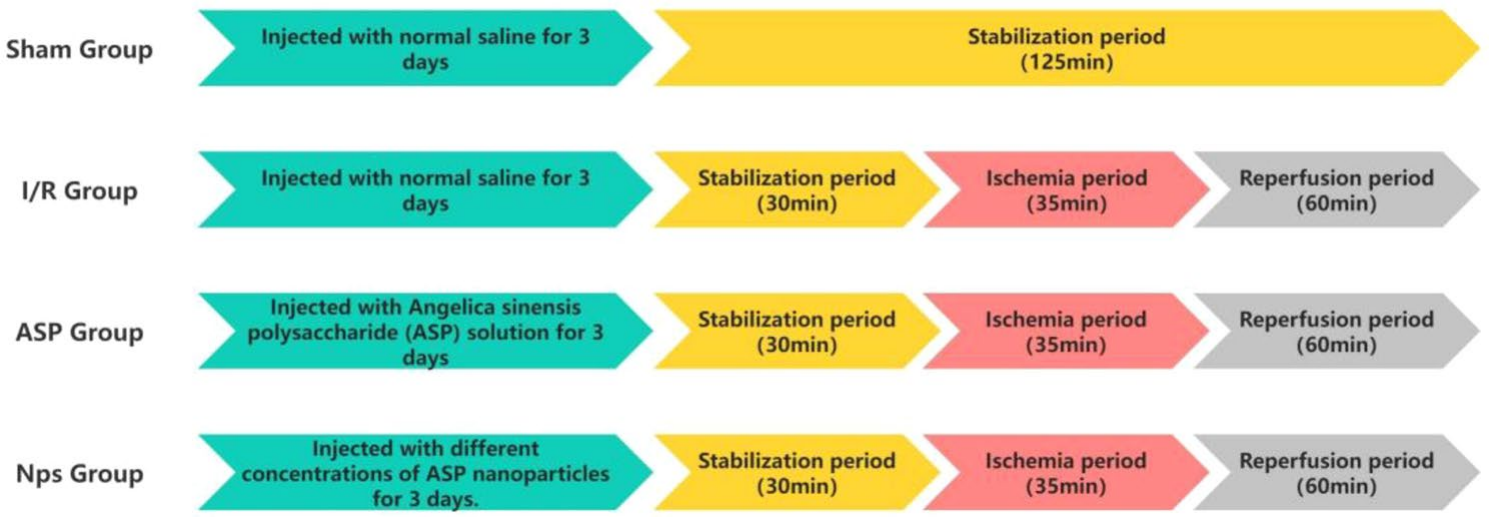
Schematic diagram of the perfusion protocol used in the Langendorff isolated heart experiment

We established the MI/RI model using the Langendorff isolated heart perfusion system. The perfusion protocols for each group are shown in Fig. 3. During the isolated heart perfusion, cardiac function was continuously monitored by inserting a Langendorff balloon into the left ventricle and connecting it to a PowerLab data acquisition system.

**Fig. 4:**
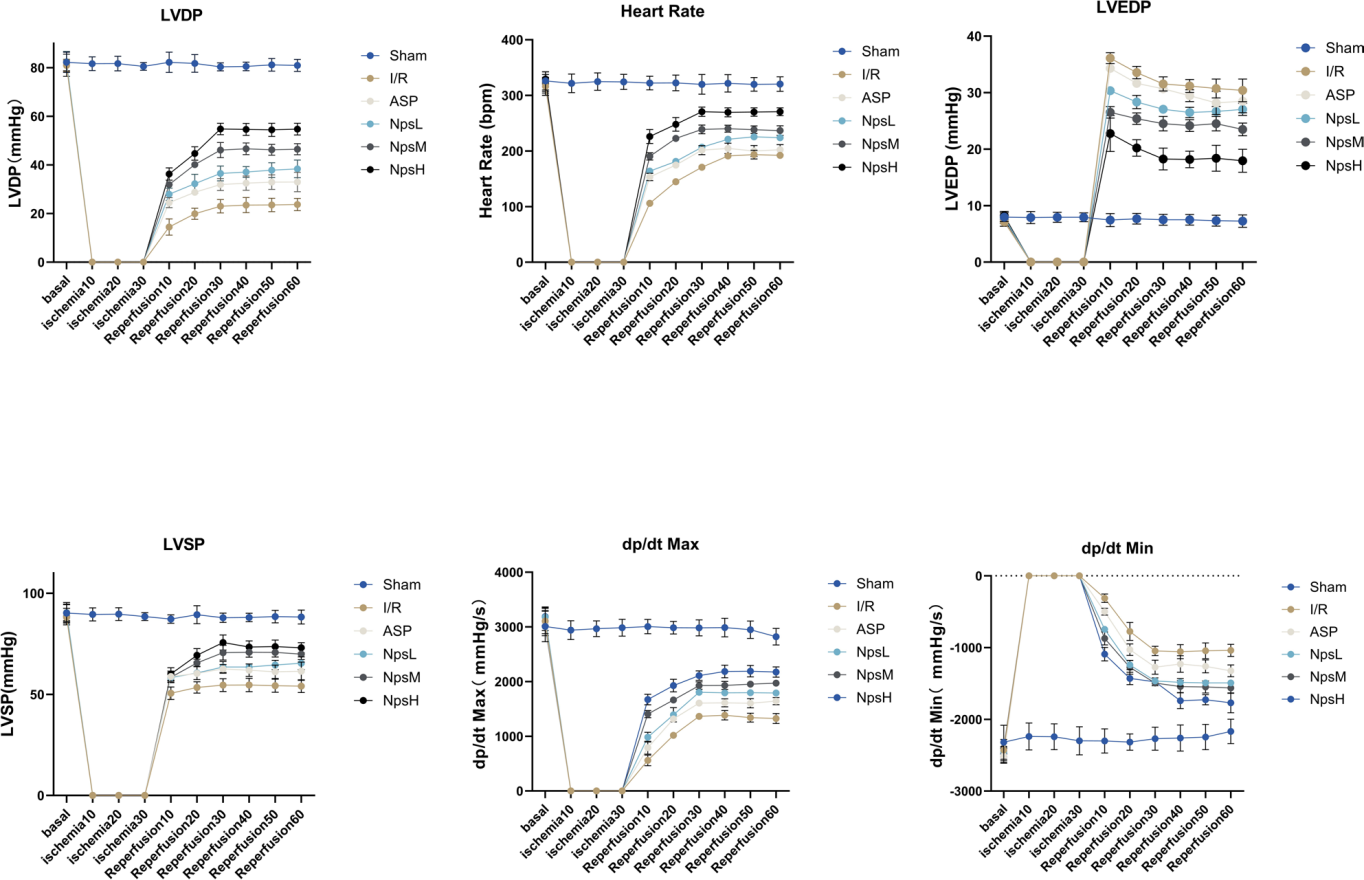
ASP@PLGA-PEG nanoparticles improve cardiac function parameters in isolated hearts following MI/RI. (**A**) Time-course changes in left ventricular developed pressure (LVDP) during the stabilization period, ischemia, and reperfusion. (**B**) Time-course changes in heart rate (HR) (**C**) Time-course changes in left ventricular end-diastolic pressure (LVEDP). (**D**) Time-course changes in left ventricular systolic pressure (LVSP). (**E**) Time-course changes in the maximum rate of pressure rise (dp/dt_Max_). (**F**) Time-course changes in the maximum rate of pressure decline (dp/dt_Min_). All data are expressed as mean ± SD (*n* = 5 per group). Statistical analysis was performed using one-way ANOVA followed by Tukey’s post hoc test

**Fig. 5:**
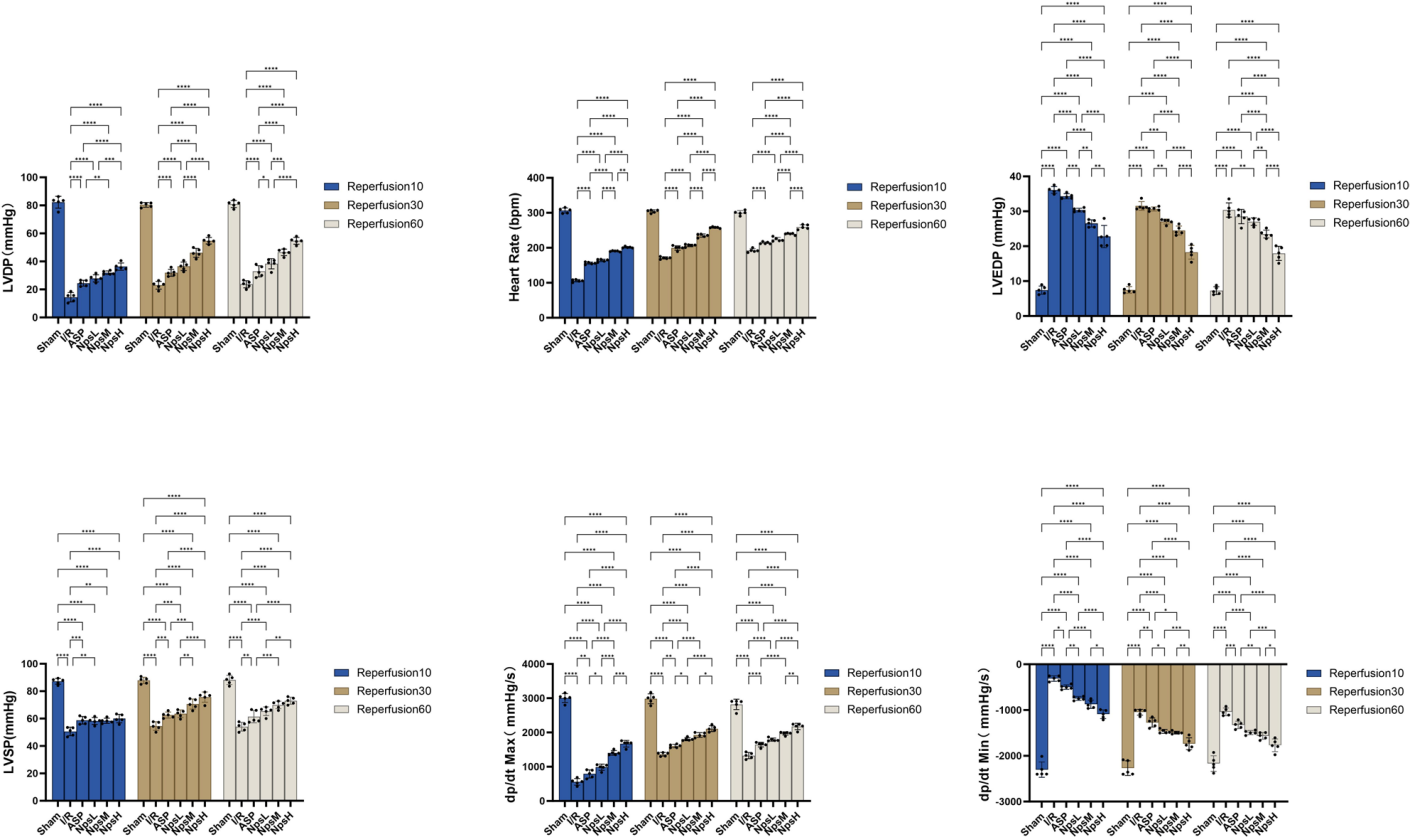
Statistical comparison of cardiac function parameters at different reperfusion time points in isolated hearts subjected to MI/RI. (**A**) Left ventricular developed pressure (LVDP) at 10, 30, and 60 minutes of reperfusion in each group. (**B**) Heart rate (HR) at 10, 30, and 60 minutes of reperfusion. (**C**) Left ventricular end-diastolic pressure (LVEDP) at 10, 30, and 60 minutes of reperfusion. (**D**) Left ventricular systolic pressure (LVSP) at 10, 30, and 60 minutes of reperfusion. (**E**) Maximum rate of left ventricular pressure rise (dp/dt_Max_). (**F**) Maximum rate of left ventricular pressure decline (dp/dtMin). Blue, brown, and grey bars represent values at reperfusion 10, 30, and 60 minutes, respectively. Data are presented as mean ± SD (*n* = 5 per group). Statistical significance: ns, not significant; ns, not significant. *p < 0.05, **p < 0.01, ***p < 0.001, ****p < 0.0001. Statistical analysis was performed using one-way ANOVA followed by Tukey’s post hoc test

Indicators of isolated cardiac function are presented in Figs. 4 and 5. Figure 4 illustrates the dynamic trends of functional parameters throughout the Langendorff perfusion process, whereas Figure 5 displays the statistical comparisons of these parameters at selected reperfusion time points. During the stabilization period, no significant differences in cardiac parameters were observed among the groups (Additional File 1: Figure S1), confirming comparable baseline cardiac function.

During Langendorff isolated heart perfusion, changes in hemodynamic parameters are shown in Fig. 4. LVDP, defined as the difference between left ventricular systolic and end-diastolic pressures, reflects myocardial contractile performance; higher LVDP values indicate stronger contractility. The mean LVDP during equilibration was 81.6 ± 3.3 mmHg. Following 35 min of global ischemia, LVDP markedly declined, reaching 14.4 ± 3.3 mmHg at 10 min of reperfusion in the I/R group. ASP preconditioning (30 mg/kg) partially improved LVDP to 32.9 ± 4.0 mmHg at 60 min of reperfusion, whereas high-dose ASP@PLGA-PEG nanoparticles (20 mg/kg) further enhanced recovery, with LVDP reaching 54.7 ± 2.4 mmHg.

Consistently, dp/dt_max (an index of systolic function) and dp/dt_min (an index of diastolic relaxation) displayed similar improvements. At 60 min of reperfusion, dp/dt_max increased from 1325.7 ± 90.6 mmHg/s in the I/R group to 2174.4 ± 93.0 mmHg/s in the NpsH group, whereas dp/dt_min improved from −1038.6 ± 84.7 mmHg/s to −1769.4 ± 138.2 mmHg/s.

Collectively, these data indicate that high-dose ASP@PLGA-PEG nanoparticles markedly enhance the recovery of both systolic and diastolic cardiac function following MI/R.

**Fig. 6:**
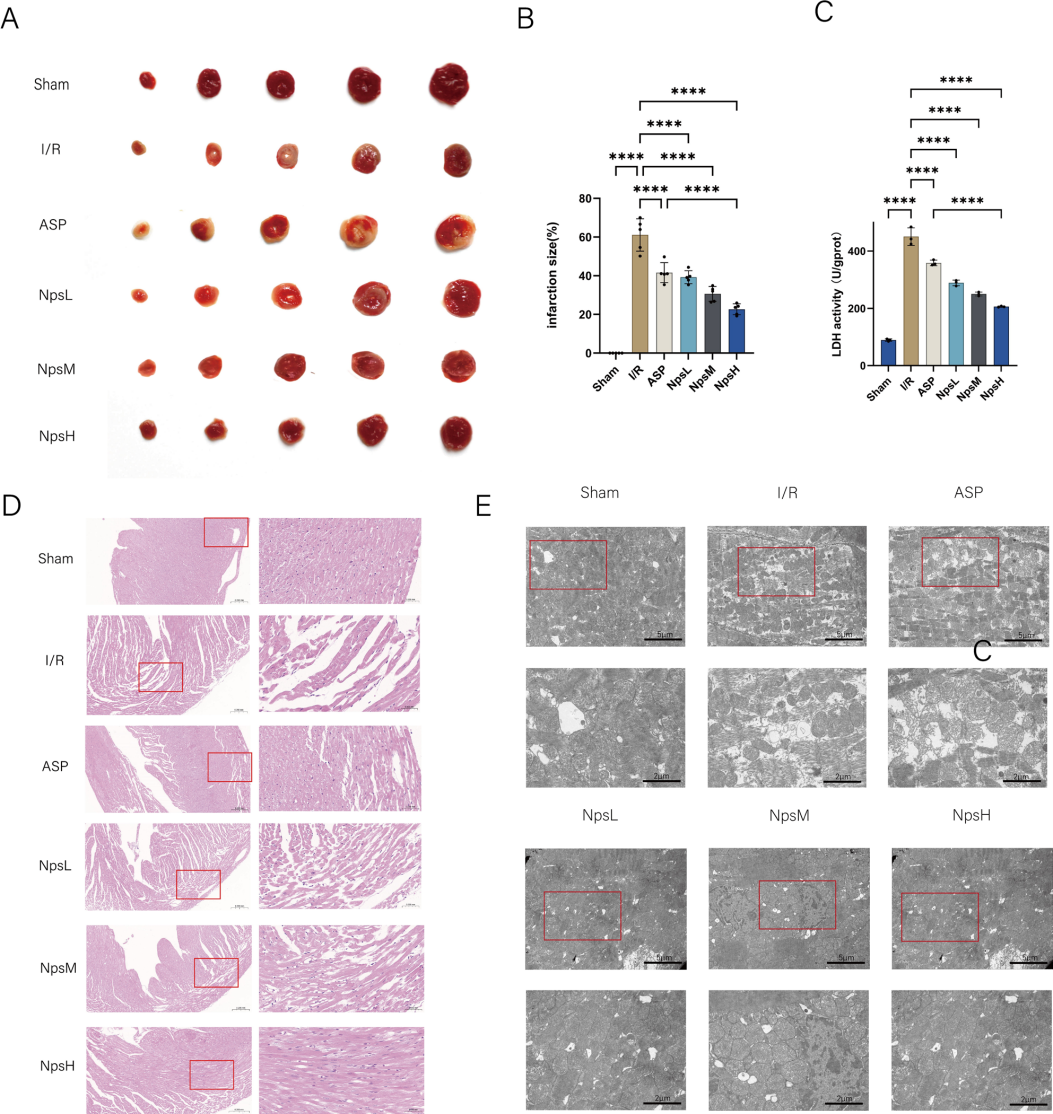
ASP@PLGA-PEG nanoparticles reduce infarct size, attenuate myocardial tissue damage, and preserve mitochondrial morphology in isolated hearts following MI/RI. (**A**) Representative TTC-stained heart slices from each group, showing viable myocardium in red and infarcted tissue in pale white, *n* = 5. (**B**) Quantification of infarct size ratio (%) in different groups, *n* = 5. (**C**) Lactate dehydrogenase (LDH) activity as an indicator of myocardial injury, *n* = 5. (**D**). Representative hematoxylin and eosin (H&E) staining of myocardial tissue sections from each group. The right panels show enlarged regions from the boxed areas, highlighting histopathological changes such as myocardial fiber integrity and inflammatory infiltration. (**E**) Transmission electron microscopy (TEM) images showing mitochondrial ultrastructure in myocardial tissues from each group. Red boxed areas indicate regions of interest; scale bars are shown in each image. *p < 0.05, **p < 0.01, ***P < 0.001, ****P < 0.0001. Statistical analysis was performed using one-way ANOVA followed by Tukey’s post hoc test

The infarct size ratio is a key indicator for evaluating the severity of myocardial injury. TTC staining was used to distinguish viable myocardium (red) from infarcted tissue (pale), as shown in Figs. 6A and 6B. In the I/R group, the infarct size reached 61%, whereas it was significantly reduced to 22.6% in the NpsH group, indicating a pronounced cardioprotective effect of ASP@PLGA-PEG during MI/R injury.

Histopathological changes in ischemic myocardium were assessed by H&E staining (Fig. 6C). The I/R group exhibited extensive necrosis, disrupted myocardial architecture, and marked inflammatory cell infiltration, while ASP@PLGA-PEG–treated hearts displayed largely preserved myocardial morphology with markedly less inflammatory infiltration.

Mitochondria play a central role in MI/R injury and ferroptosis, an iron-dependent form of cell death driven by lipid peroxidation. To further examine mitochondrial ultrastructure, TEM analysis was performed (Fig. 6D). In the I/R group, mitochondria exhibited severe swelling, cristae disruption, and vacuolization. In contrast, mitochondria from the ASP@PLGA-PEG group retained more intact cristae and showed substantially reduced vacuolization.

### ASP@PLGA-PEG nanoparticles suppress oxidative stress in isolated hearts by activating ATF6 and inhibiting ER stress

**Fig. 7:**
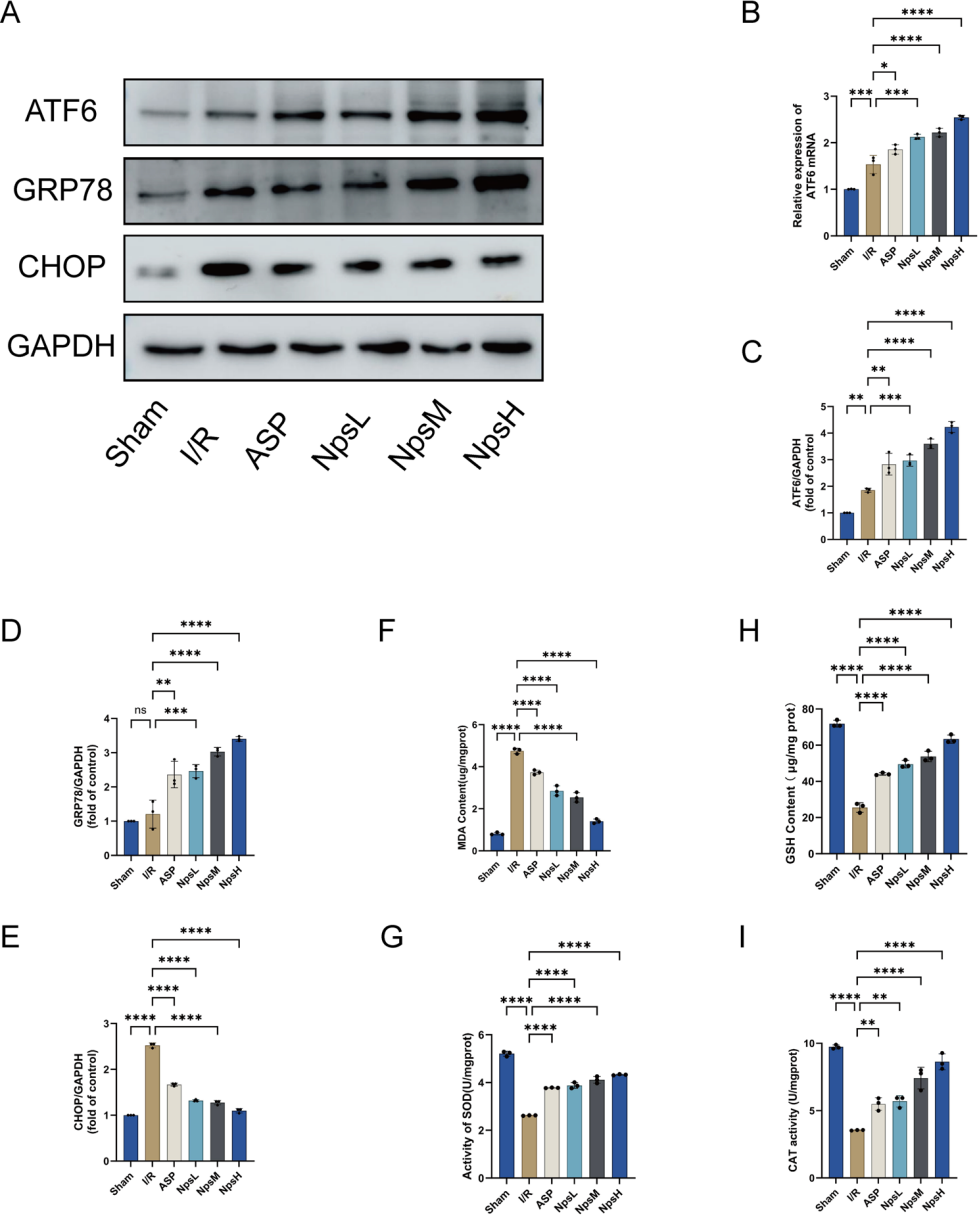
Representative effects of ASP and ASP@PLGA-PEG nanoparticles on ATF6-mediated ER stress response and oxidative stress in isolated hearts. (**A**) Representative Western blot bands showing protein expression levels of ATF6, GRP78/BIP, CHOP, and GAPDH in myocardial tissues from different groups. (**B**) Relative mRNA expression levels of ATF6 detected by qRT-PCR. (**C**) Quantitative analysis of ATF6 protein expression normalized to GAPDH. (**D**) Quantitative analysis of GRP78/BIP protein expression normalized to GAPDH. (**E**) Quantitative analysis of CHOP protein expression normalized to GAPDH. (**F**) Malondialdehyde (MDA) content as an indicator of lipid peroxidation. (**G**) Superoxide dismutase (SOD) activity. (**H**) Reduced glutathione. (GSH) content. (**I**) Catalase (CAT) activity. All data are presented as mean ± SD (*n* = 3 per group). *p < 0.05, **p < 0.01, ***p < 0.001, ****p < 0.0001. Statistical analysis was performed using one-way ANOVA followed by Tukey’s post hoc test

In our previous studies, ASP was shown to activate ATF6 to suppress endoplasmic reticulum (ER) stress and thereby ameliorate MI/R injury [17, 18]. In this study, this effect was further confirmed in the Langendorff isolated heart perfusion model. As shown in Fig. 7B, ASP significantly upregulated ATF6 mRNA expression compared with the I/R group, and high-dose ASP@PLGA-PEG nanoparticles further enhanced ATF6 mRNA expression relative to free ASP. Western blot analysis (Fig. 7A, C–E) demonstrated that high-dose ASP@PLGA-PEG markedly increased ATF6 protein expression. In addition, GRP78/BIP, an upstream ER stress sensor, was also significantly upregulated, whereas CHOP—a marker of irreversible terminal ER stress—was significantly downregulated. These data indicate that ASP@PLGA-PEG nanoparticles more effectively activate ATF6 signaling and enhance the adaptive unfolded protein response, thereby attenuating excessive ER stress.

Because oxidative stress is a critical regulator of both cardiomyocyte apoptosis and ferroptosis, we next evaluated oxidative stress status following MI/R and the antioxidative effects of ASP@PLGA-PEG nanoparticles. Oxidative and antioxidative biomarkers (MDA, SOD, CAT, and GSH) are presented in Fig. 7F–I. Compared with the Sham group, MDA—the end-product of lipid peroxidation—was significantly elevated in the I/R group, whereas ASP and ASP@PLGA-PEG both markedly reduced MDA accumulation. Moreover, antioxidant defense indicators (SOD, CAT, and GSH) were significantly restored following treatment, with ASP@PLGA-PEG showing a superior antioxidative effect compared with free ASP.

### ASP@PLGA-PEG nanoparticles inhibit ferroptosis in MI/RI by blocking NCOA4-mediated ferritinophagy

**Fig. 8:**
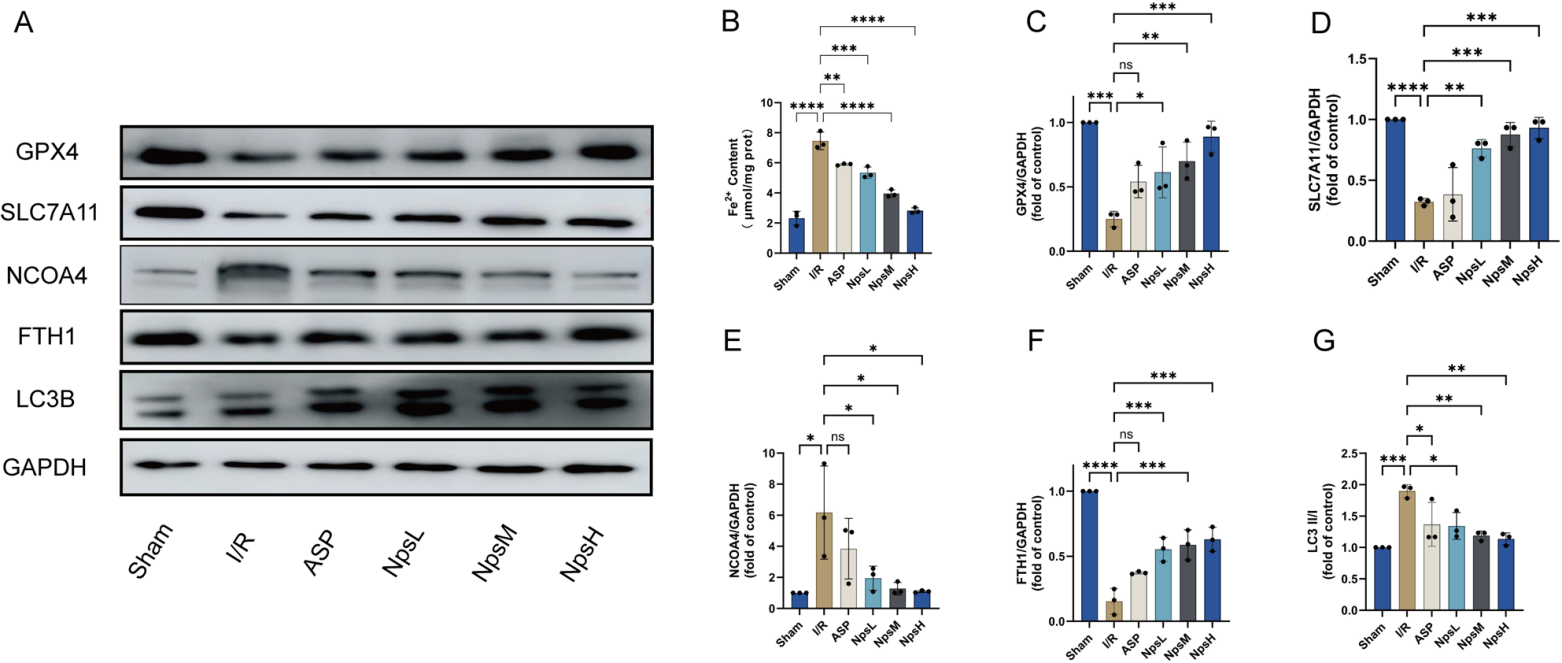
ASP@PLGA-PEG nanoparticles inhibit ferroptosis by blocking NCOA4-mediated ferritinophagy in MI/RI. (**A**) Representative Western blot images showing the protein expression of GPX4, SLC7A11, NCOA4, FTH1, LC3B, and GAPDH in myocardial tissues from each group. (**B**) Quantification of Fe^2 +^ content in myocardial tissues. (**C**) Densitometric analysis of GPX4 protein expression normalized to GAPDH. (**D**) Densitometric analysis of SLC7A11 protein expression normalized to GAPDH. (**E**) Densitometric analysis of NCOA4 protein expression normalized to GAPDH. (**F**) Densitometric analysis of FTH1 protein expression normalized to GAPDH. (**G**) LC3-II/I ratio indicating autophagic activity related to ferritinophagy. All data are presented as mean ± SD (*n* = 3 per group). *p < 0.05, **p < 0.01, ***p < 0.001, ****p < 0.0001. Statistical analysis was performed using one-way ANOVA followed by Tukey’s post hoc test

TEM analysis demonstrated that ASP@PLGA-PEG nanoparticles improved mitochondrial morphology in mouse cardiomyocytes. Ferroptosis, an iron-dependent lipid peroxidation–driven cell death process, is characterized by GPX4 inactivation and iron overload. Intracellular Fe^2 +^ content quantified by microcolorimetry (Fig. 8B) showed that Fe^2 +^ was markedly increased in the I/R group compared with Sham. Pretreatment with low-dose ASP@PLGA-PEG reduced Fe^2 +^ accumulation, whereas high-dose ASP@PLGA-PEG significantly suppressed Fe^2 +^ elevation. Western blot analysis (Fig. 8A, C, D) further revealed that GPX4 and SLC7A11 protein levels were profoundly decreased in the I/R group, while ASP@PLGA-PEG pretreatment prevented these reductions, consistent with its antioxidative and anti-ferroptotic effects.

To further determine whether ferritinophagy was involved, NCOA4 expression was assessed (Fig. 8A, E). NCOA4 protein was strongly upregulated in the I/R group and was significantly inhibited following ASP@PLGA-PEG pretreatment. Since NCOA4 binds FTH1 and mediates its autophagic degradation, we next examined FTH1 and LC3B (Fig. 8A, F, G). Compared with Sham, the I/R group exhibited markedly lower FTH1 expression and a significant increase in LC3-II formation, indicating enhanced NCOA4-dependent ferritinophagic flux. ASP@PLGA-PEG pretreatment reduced LC3-I to LC3-II conversion and restored FTH1 levels, suggesting that ASP@PLGA-PEG suppresses ferritinophagy activation during MI/R injury.

### The protection of ASP@PLGA-PEG on heart function of MI/RI mice

**Fig. 9:**
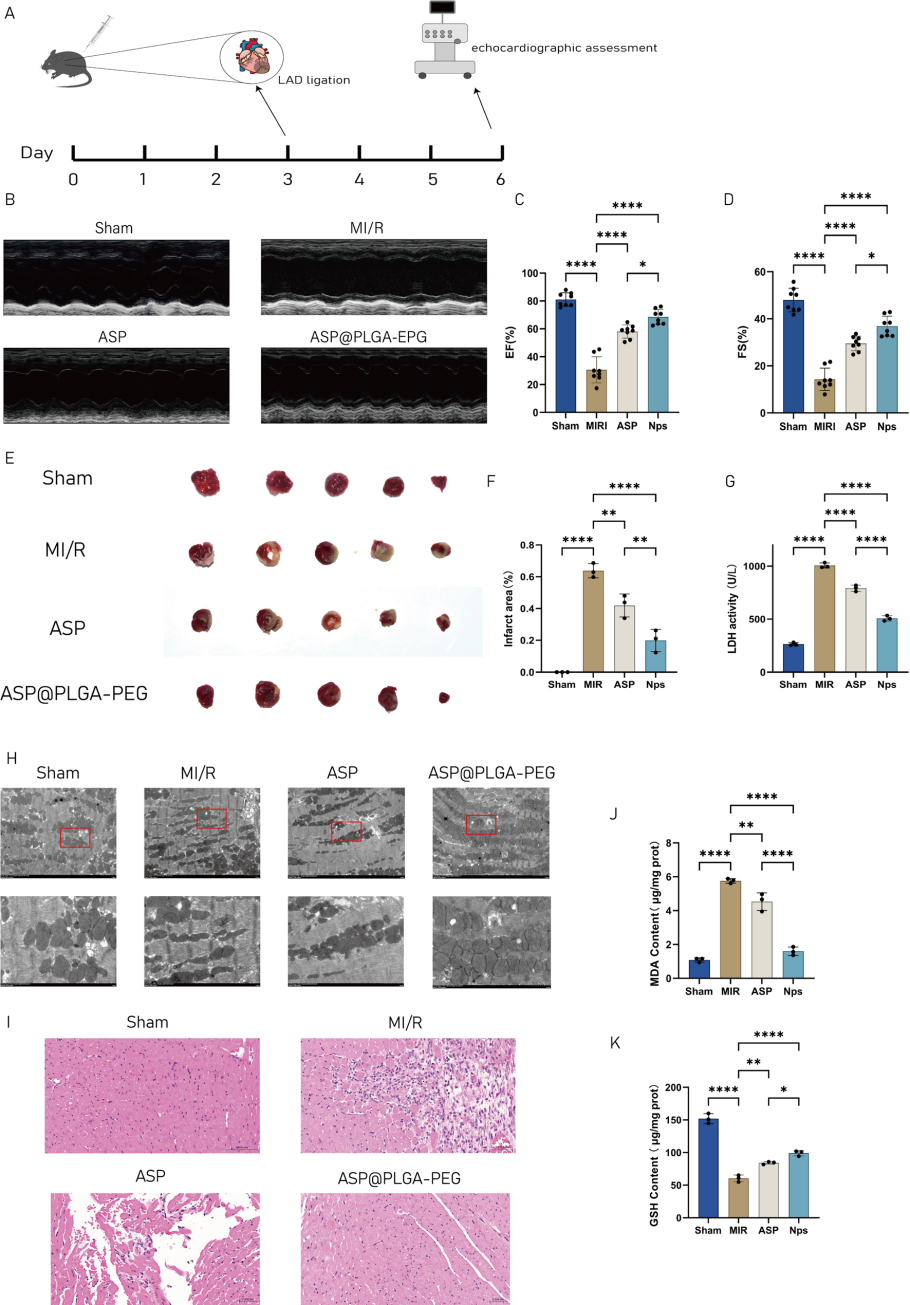
Evaluation of the cardioprotective effects of ASP@PLGA-PEG in a mouse MI/R model. (**A**) Schematic of the MI/R experimental protocol. (**B–D**) Echocardiographic assessment of cardiac systolic function (EF% and FS%) in different groups, *n* = 8. (**E, F**) Representative TTC staining images and quantification of myocardial infarct size, *n* = 3. (**G**) Serum LDH activity in different groups, *n* = 3. (**H**) Representative TEM images of myocardial ultrastructure. (**I**) Representative HE staining of myocardial tissue. (**J, K**) Quantification of oxidative stress markers (MDA and GSH) in myocardial tissues, *n* = 3. *p < 0.05, **p < 0.01, ***p < 0.001, ****p < 0.0001. Statistical analysis was performed using one-way ANOVA followed by Tukey’s post hoc test

To further determine whether the enhanced anti-ferroptotic effects of ASP@PLGA-PEG translate

into improved cardioprotection in vivo, cardiac functional and structural indices were subsequently evaluated in the mouse MI/R model (Fig. 9). As shown in Fig. 9A, mice were pre-treated with ASP or ASP@PLGA-PEG for 3 days prior to LAD ligation–reperfusion to establish the MI/R model.

Echocardiographic assessment (Fig. 9B–D) demonstrated that MI/R markedly reduced EF% and FS%, indicating severe impairment of cardiac systolic function. ASP treatment partially improved these parameters, whereas ASP@PLGA-PEG led to a more pronounced recovery, suggesting that

nanoparticle encapsulation significantly enhances the functional benefits of ASP. TTC staining revealed substantial infarct expansion in MI/R mice (Fig. 9E–F). ASP reduced infarct size, while

ASP@PLGA-PEG further diminished infarcted area to a markedly greater extent. Serum LDH activity (Fig. 9G), a biochemical indicator of myocardial injury, was dramatically elevated in MI/R mice, whereas both ASP and ASP@PLGA-PEG attenuated this elevation, with the nanoparticle formulation showing superior efficacy.

Transmission electron microscopy (Fig. 9H) confirmed profound ultrastructural damage in MI/R hearts, characterized by mitochondrial swelling and cristae disruption. ASP alleviated these abnormalities to some extent, whereas ASP@PLGA-PEG almost normalized mitochondrial morphology. Histological evaluation by H&E staining (Fig. 9I) yielded consistent results, with ASP@PLGA-PEG more effectively reducing inflammatory cell infiltration and structural deterioration.

Furthermore, oxidative stress analysis (Fig. 9J–K) showed significantly increased MDA and decreased GSH levels in MI/R hearts, whereas ASP treatment partially restored redox homeostasis. Importantly, ASP@PLGA-PEG produced a markedly greater reduction in MDA and elevation in GSH,

indicating a stronger antioxidative effect.

Collectively, these data demonstrate that ASP@PLGA-PEG provides substantially enhanced cardioprotection compared with free ASP, manifested by improved cardiac function, reduced infarct size, attenuated tissue injury, and more effective suppression of oxidative stress in MI/R injury.

**Fig. 10:**
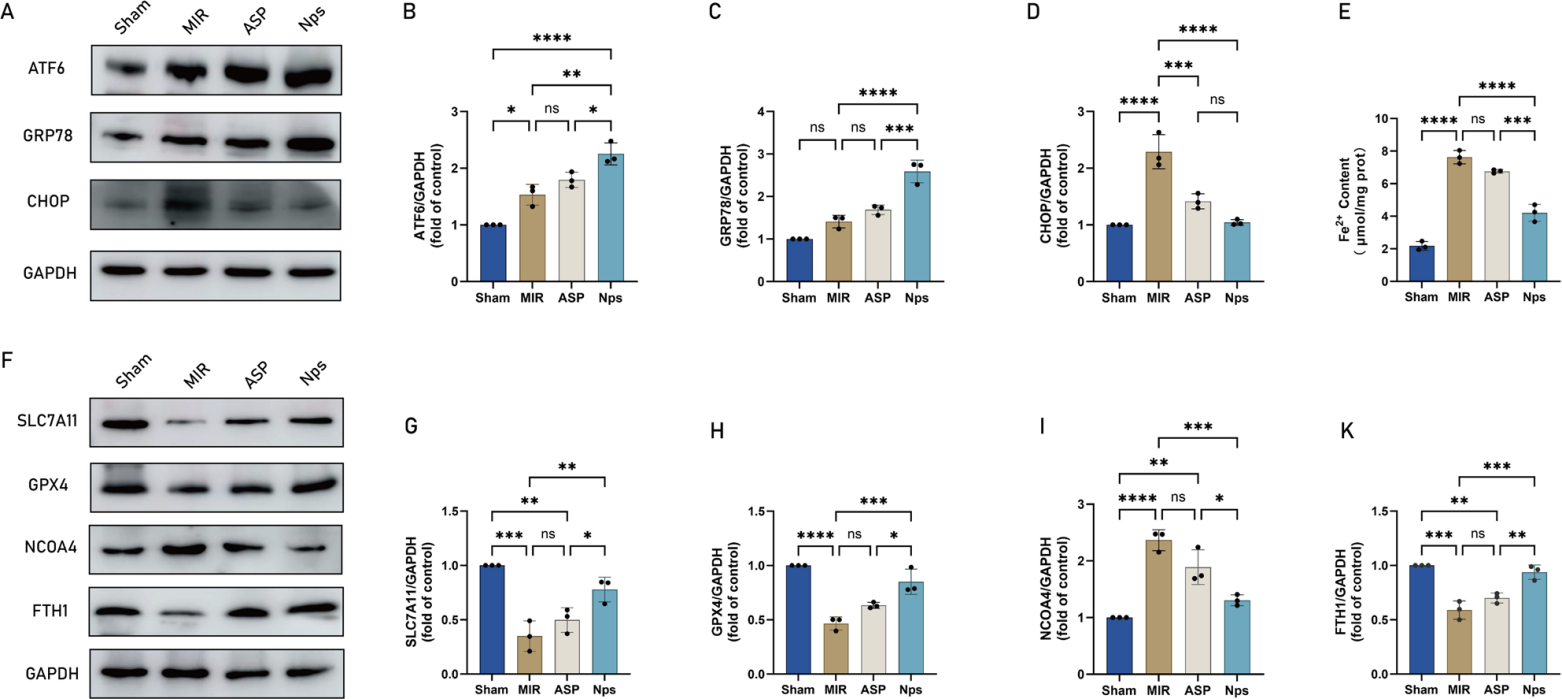
ASP@PLGA-PEG more effectively inhibits ER stress and ferritinophagy-associated ferroptosis in MI/R hearts. (**A–D**) Representative Western blot bands showing protein expression levels of ATF6, GRP78/BIP, CHOP, and GAPDH in myocardial tissues from different groups. (**E**) Quantification of Fe2 + content in myocardial tissues. (**F–K**) Representative Western blot bands showing protein expression levels of SLC7A11, GPX4, NCOA4, FTH1, and GAPDH in myocardial tissues from different groups. *N* = 3 per group. *p < 0.05, **p < 0.01, ***p < 0.001, ****p < 0.0001. Statistical analysis was performed using one-way ANOVA followed by Tukey’s post hoc test

To further examine whether ASP@PLGA-PEG modulates ATF6-dependent signaling in vivo, we assessed ER stress and ferroptosis-related markers in myocardial tissues (Fig. 10). MI/R markedly activated ER stress, reflected by significant increases in ATF6, GRP78, and CHOP protein expression, accompanied by a substantial elevation in myocardial Fe^2 +^ levels (Fig. 10A–E). Fe^2 +^ accumulation is a representative biochemical indicator of ferroptosis, and importantly, TEM observations in MI/R myocardium demonstrated typical ferroptotic ultrastructural features (increased mitochondrial membrane density, reduced or lost cristae, and mitochondrial shrinkage). These observations provide a mechanistic rationale for further linking ferroptosis and ferritinophagy. Consistent with this, MI/R downregulated SLC7A11 and GPX4 and upregulated NCOA4 (Fig. 10F–K), indicating enhanced ferritinophagy-driven ferroptotic sensitivity. ASP partially reversed these alterations, whereas ASP@PLGA-PEG exerted a more pronounced regulatory effect, showing greater suppression of ATF6–CHOP signaling, lower Fe^2 +^ accumulation, reduced NCOA4, and more robust restoration of GPX4, SLC7A11, and FTH1. In summary, ASP@PLGA-PEG more effectively suppressed the ATF6–NCOA4–ferritinophagy axis and ferroptosis than free ASP, providing a mechanistic basis for its greater cardioprotective efficacy.

### Protective effect of ASP@PLGA-PEG on oxygen and glucose deprivation and reoxygenation (OGD/R) injured HL-1 cells

To further validate these findings at the cellular level, we next examined the protective effects of

**Fig. 11:**
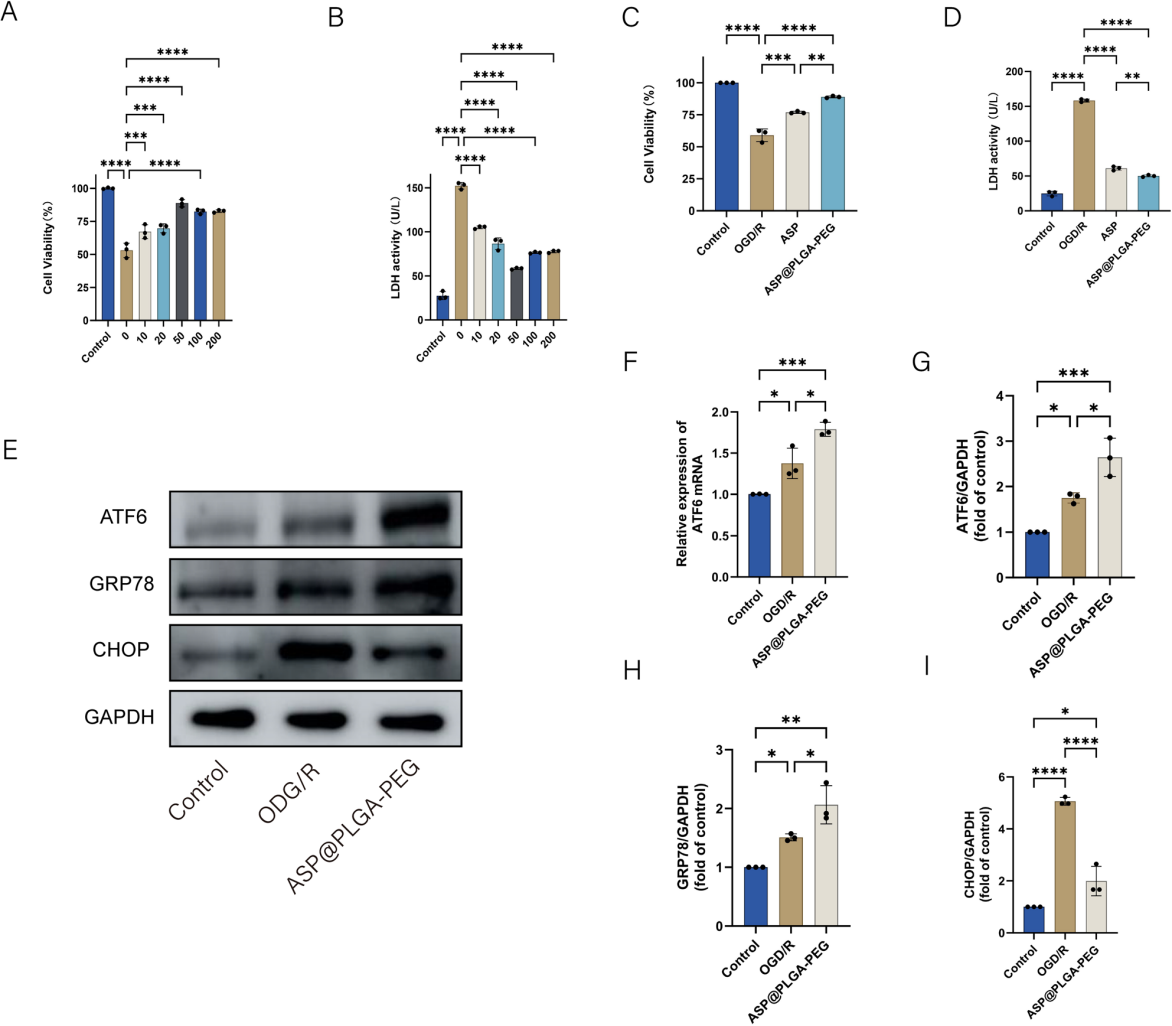
Protective effect of ASP@PLGA-PEG nanoparticles against OGD/R-induced injury in HL-1 cardiomyocytes and its regulation of the ATF6 pathway. (**A**) Cell viability of HL-1 cardiomyocytes treated with different concentrations of ASP@PLGA-PEG nanoparticles (10, 20, 50, 100, and 200 μg/mL) under OGD/R conditions, assessed by the CCK-8 assay. (**B**) LDH activity in the culture supernatant of HL-1 cardiomyocytes treated with various concentrations of ASP@PLGA-PEG nanoparticles under OGD/R conditions. (**C**) Comparison of cell viability between the ASP and ASP@PLGA-PEG groups (both at 50 μg/mL) after OGD/R injury. (**D**) Comparison of LDH activity between the ASP and ASP@PLGA-PEG groups after OGD/R injury. (**E**) Representative Western blot bands showing the protein expression levels of ATF6, GRP78, and CHOP in HL-1 cardiomyocytes in the control, OGD/R, and ASP@PLGA-PEG groups. (**F**) Relative expression of ATF6 mRNA detected by qRT-PCR. (**G–I**) Quantification of ATF6 (**G**), GRP78 (**H**), and CHOP (**I**) protein levels normalized to GAPDH. *n* = 3, *p < 0.05, **p < 0.01, ***p < 0.001, ****p < 0.0001. Statistical analysis was performed using one-way ANOVA followed by Tukey’s post hoc test

ASP@PLGA-PEG in HL-1 cardiomyocytes subjected to OGD/R injury (Fig. 11). Cell viability after OGD/R injury was assessed using the CCK-8 assay, and LDH release was measured to evaluate the extent of cellular damage. To identify the optimal protective concentration of ASP@PLGA-PEG nanoparticles, various concentrations (10, 20, 50, 100, and 200 μg/mL) were tested. As shown in Fig. 11A, OGD/R injury markedly reduced HL-1 cell viability to nearly half of the control level, while pretreatment with ASP@PLGA-PEG nanoparticles significantly restored cell viability in a concentration-dependent manner. Among the tested concentrations, 50 μg/mL provided the greatest protective effect, with cell viability improving close to baseline levels. Consistent with this, LDH activity in the culture supernatant showed that cells pretreated with 50 μg/mL ASP@PLGA-PEG released the lowest amount of LDH, indicating reduced membrane damage and better cellular integrity (Fig. 11B). Therefore, 50 μg/mL was selected as the working concentration for subsequent experiments.

To compare the protective effects of ASP@PLGA-PEG nanoparticles with free ASP, HL-1 cardiomyocytes were pretreated with either ASP or ASP@PLGA-PEG at the same concentration. Following OGD/R injury, cells treated with ASP@PLGA-PEG showed higher viability than those treated with free ASP (Fig. 11C). Likewise, LDH activity was lower in the ASP@PLGA-PEG group (Fig. 11D), confirming superior cytoprotection of the nanoparticle formulation.

At the mechanistic level, ATF6 mRNA expression increased after OGD/R injury and was further upregulated in the ASP@PLGA-PEG group compared with free ASP (Fig. 11F). Western blot analysis (Fig. 11E, G–I) showed that ATF6 and GRP78 protein expression levels were also elevated in the ASP@PLGA-PEG group, while CHOP expression—representing sustained and irreversible ER stress—was markedly reduced. These findings indicate that ASP@PLGA-PEG nanoparticles enhance ATF6 activation and improve the adaptive unfolded protein response, thereby attenuating excessive ER stress and protecting cardiomyocytes from OGD/R-induced injury.

### ASP@PLGA-PEG nanoparticles alleviate oxidative stress

**Fig. 12:**
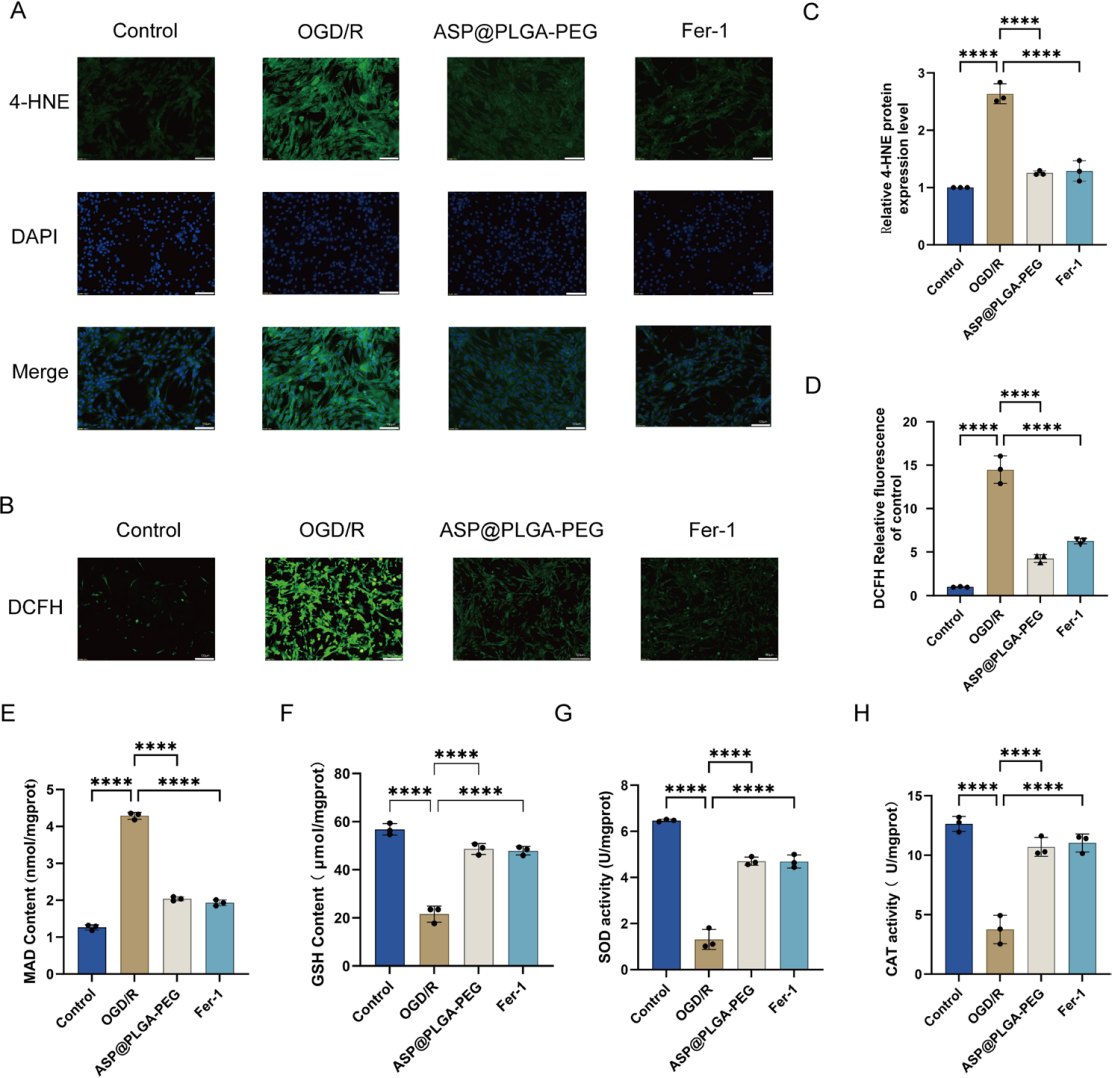
ASP@PLGA-PEG nanoparticles attenuate oxidative stress in HL-1 cardiomyocytes subjected to OGD/R injury. (**A**) Representative immunofluorescence images showing 4-HNE expression (green) and DAPI-stained nuclei (blue) in HL-1 cardiomyocytes under control, OGD/R, ASP@PLGA-PEG, and Ferrostatin-1 (fer-1) treatment conditions. Scale bar = Xμm. (**B**) Representative DCFH-DA fluorescence images indicating intracellular ROS levels in HL-1 cardiomyocytes under the indicated treatments. Scale bar = Xμm. (**C**) Quantification of relative 4-HNE protein expression. (**D**) Quantification of DCFH fluorescence intensity as a measure of intracellular ROS accumulation. (**E–H**) Biochemical analysis of oxidative stress markers: MDA content (**E**), GSH content (**F**), SOD activity (**G**), and CAT activity (**H**) in HL-1 cardiomyocytes. *n*=3, *p<0.05, **p<0.01, ***p<0.001, ****p<0.0001. Statistical analysis was performed using one-way ANOVA followed by Tukey’s post hoc test

Given that ASP@PLGA-PEG activated ATF6 and alleviated ER stress in HL-1 cells (Fig. 12), we next examined whether it also mitigates OGD/R-induced oxidative stress (Fig. 12). CAT, SOD, MDA, and 4-hydroxy-2-nonenal (4-HNE) are commonly used biomarkers for evaluating oxidative stress in cells and tissues. After OGD/R injury, HL-1 cardiomyocytes showed a significant decrease in CAT and SOD activity and a marked increase in MDA levels (Fig. 12E, G, H). In addition, 4-HNE fluorescence intensity was markedly elevated following OGD/R (Fig. 12A, C). Pretreatment with ASP@PLGA-PEG or Ferrostatin-1 effectively reversed these changes, indicating reduced oxidative damage. ROS accumulation, a hallmark of both ER stress and ferroptosis, was also significantly increased after OGD/R injury but was substantially reduced by ASP@PLGA-PEG (Fig. 12B, D). Furthermore, intracellular reduced GSH levels were significantly higher in the treatment groups compared with OGD/R alone (Fig. 12F), further supporting the antioxidative effect of ASP@PLGA-PEG nanoparticles.

### ASP@PLGA-PEG nanoparticles protect mitochondrial function and inhibit ferroptosis by suppressing NCOA4-mediated ferritinophagy

**Fig. 13:**
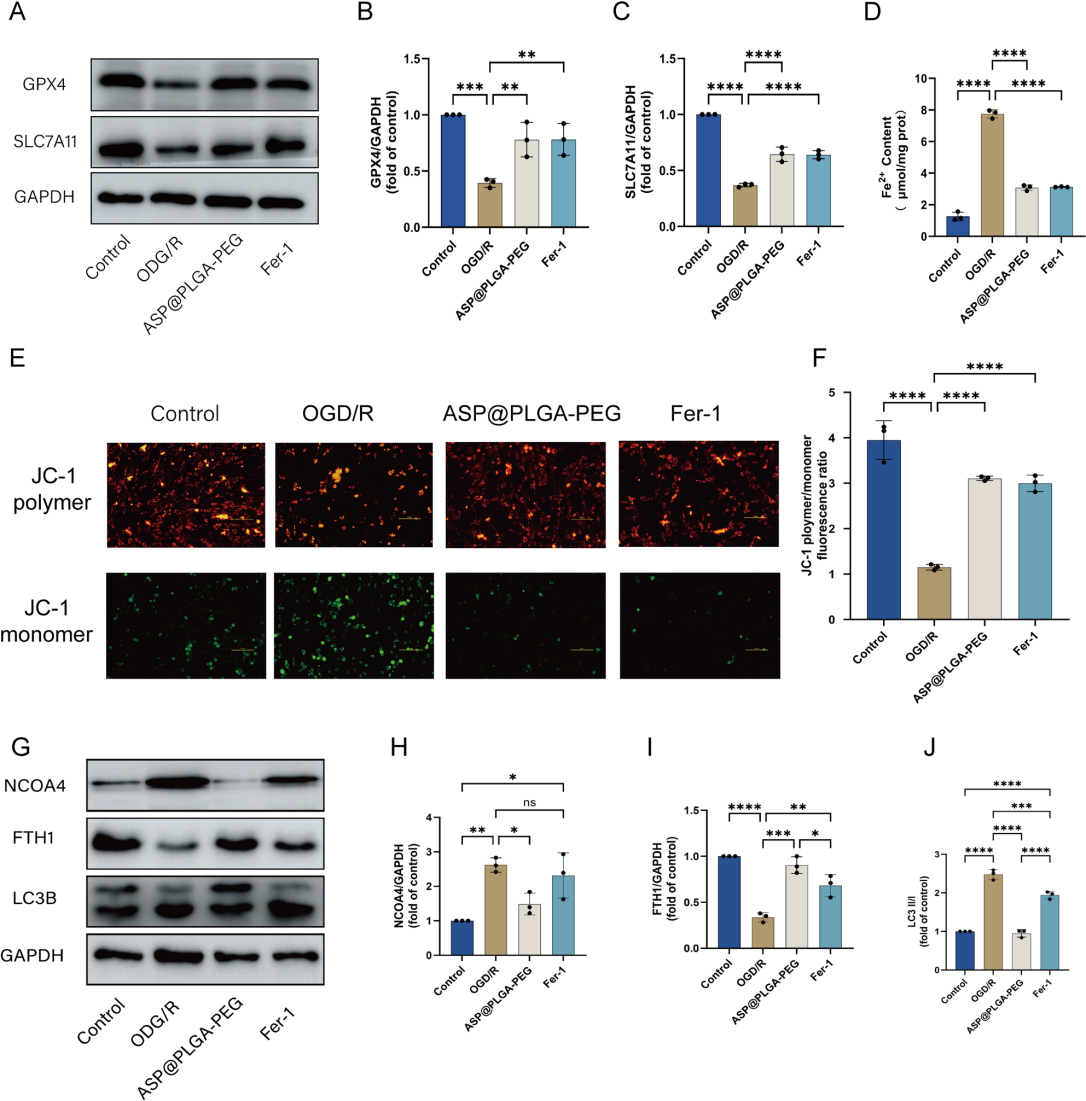
ASP@PLGA-PEG nanoparticles protect HL-1 cardiomyocytes against OGD/R-induced ferroptosis and mitochondrial dysfunction by regulating antioxidant proteins and inhibiting ferritinophagy. (**A**) Representative Western blot bands showing the protein expression levels of GPX4 and SLC7A11 in HL-1 cardiomyocytes under control, OGD/R, ASP@PLGA-PEG, and Ferrostatin-1 (fer-1) treatment conditions. (**B, C**) Quantification of GPX4 (**B**) and SLC7A11 (**C**) protein levels normalized to GAPDH. (**D**) Intracellular ferrous iron (Fe^2 +^) content measured in HL-1 cardiomyocytes under the indicated conditions. (**E**) Representative JC-1 staining images showing mitochondrial membrane potential (Δψm) changes; red fluorescence indicates JC-1 aggregates (high Δψm), and green fluorescence indicates JC-1 monomers (low Δψm). (**F**) Quantification of the JC-1 polymer-to-monomer fluorescence ratio. (**G**) Representative Western blot bands showing the expression of NCOA4, FTH1, and LC3B proteins under the indicated treatments. (**H–J**) Quantification of NCOA4 (**H**), FTH1 (**I**), and the LC3-II/I ratio (**J**) normalized to GAPDH. *n* = 3, *p < 0.05, **p < 0.01, ***p < 0.001, ****p < 0.0001. Statistical analysis was performed using one-way ANOVA followed by Tukey’s post hoc test

Mitochondrial membrane potential (Δψm) is a key indicator of mitochondrial functional integrity [27]. Oxygen acts as the terminal electron acceptor in the mitochondrial electron transport chain (ETC) and is essential for efficient ATP production. Under hypoxic conditions, ETC activity is impaired due to oxygen deficiency, resulting in decreased ATP generation and disrupted cellular energy metabolism. Consequently, the mitochondrial membrane potential gradually declines, leading to membrane depolarization [28]. JC-1 staining showed that OGD/R injury caused a marked loss of Δψm, as reflected by a shift from red JC-1 aggregates to green monomers (Fig. 13E). Pretreatment with

ASP@PLGA-PEG nanoparticles restored Δψm, similar to the effect observed with Ferrostatin-1 (Fig. 13F), indicating improved mitochondrial functional status.

To evaluate the antioxidant defense system following OGD/R injury, GPX4 and SLC7A11 expression levels were assessed. Western blot analysis revealed that GPX4 and SLC7A11 were significantly decreased in the OGD/R group, whereas ASP@PLGA-PEG pretreatment restored both protein levels (Fig. 13A–C). Consistently, intracellular ferrous iron (Fe^2 +^) content was significantly elevated after OGD/R but was markedly reduced by ASP@PLGA-PEG (Fig. 13D).

To determine whether these protective effects were associated with inhibition of ferritinophagy, NCOA4, FTH1, and LC3B were examined. NCOA4 expression was markedly increased in the OGD/R group and significantly decreased following ASP@PLGA-PEG treatment (Fig. 13G, H). FTH1 expression, which was suppressed by OGD/R, was restored by ASP@PLGA-PEG (Fig. 13I).

Moreover, the conversion of LC3-I to LC3-II was reduced in the ASP@PLGA-PEG group, indicating suppressed autophagic flux and inhibition of ferritinophagy (Fig. 13J).

Taken together, these in vitro findings indicate that ASP@PLGA-PEG nanoparticles protect HL-1 cardiomyocytes from OGD/R-induced injury by preserving mitochondrial function, enhancing the antioxidant defense system, and inhibiting NCOA4-mediated ferritinophagy.

### ATF6 knockdown abolishes the anti-ferroptotic effects of ASP@PLGA-PEG

**Fig. 14:**
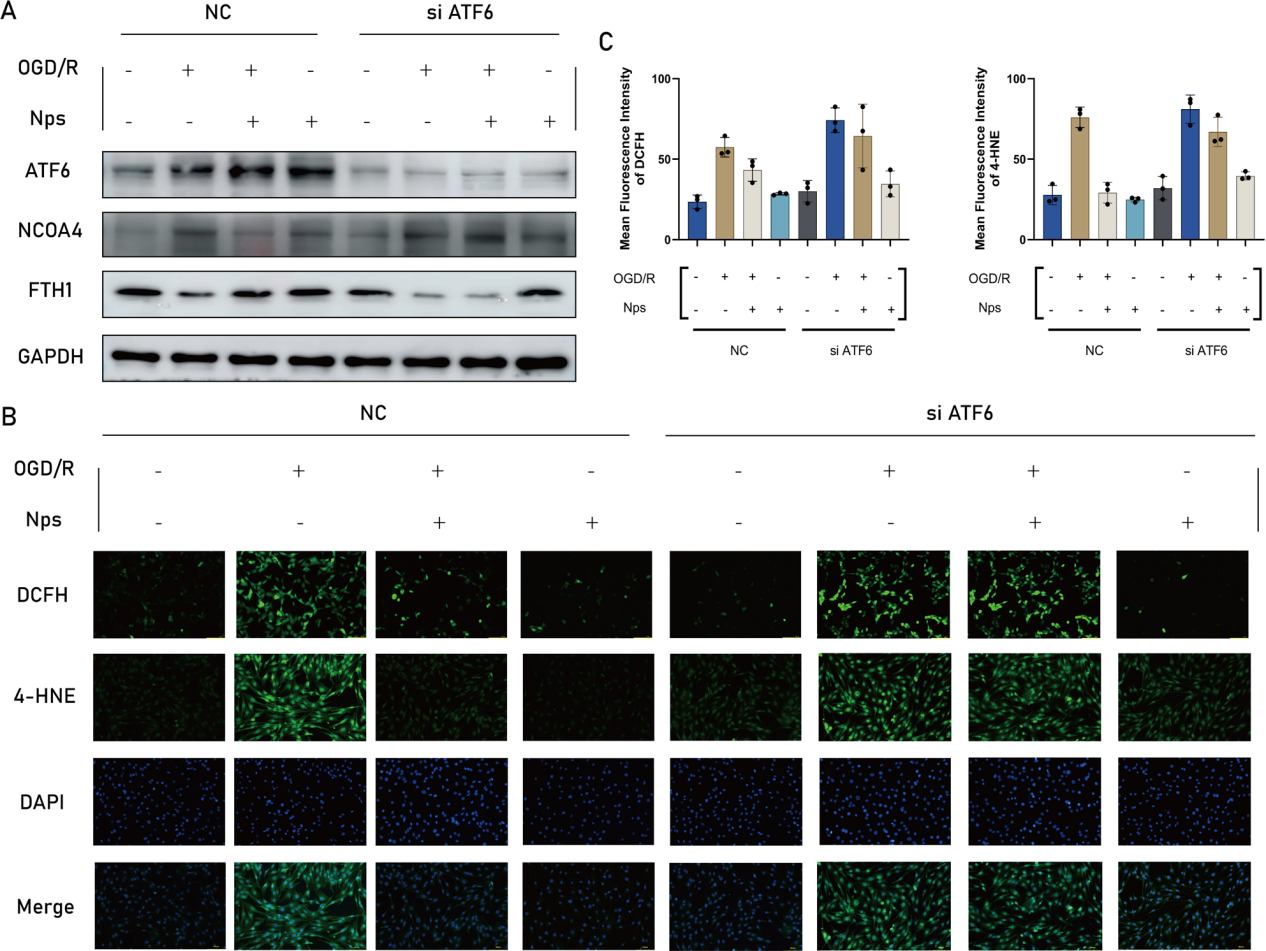
ATF6 is required for the anti-ferroptotic effect of ASP@PLGA-PEG nanoparticles in HL-1 cardiomyocytes. (**A**) Representative Western blot bands showing the protein expression levels of ATF6, NCOA4, and FTH1 in HL-1 cardiomyocytes transfected with negative control (NC) siRNA or ATF6 siRNA (siATF6), under control, OGD/R, and ASP@PLGA-PEG (nps) treatment conditions. (**B**) Representative fluorescence images of DCFH-DA and 4-HNE staining showing intracellular ROS generation and lipid peroxidation under the indicated treatments in NC and siATF6 groups. (**C**) Quantification of mean fluorescence intensity of DCFH-DA (left) and 4-HNE. (right). *n* = 3 per group. *p < 0.05, **p < 0.01, ***p < 0.001, ****p < 0.0001. Statistical analysis was performed using one-way ANOVA followed by Tukey’s post hoc test

To further determine whether the cytoprotective and anti-ferroptotic effects of ASP@PLGA-PEG are ATF6-dependent, ATF6 expression was silenced using siRNA in HL-1 cells prior to OGD/R. As shown in Fig. 14A, siATF6 markedly reduced ATF6 protein levels, and correspondingly abolished the regulatory effects of ASP@PLGA-PEG on NCOA4 and FTH1 expression. In the NC group, ASP@PLGA-PEG significantly suppressed NCOA4 and restored FTH1, consistent with ferritinophagy inhibition. However, these effects were largely lost following ATF6 knockdown, indicating that the ASP@PLGA-PEG-mediated suppression of NCOA4-dependent ferritinophagy requires intact ATF6 signaling.

Consistently, fluorescence imaging further demonstrated that the reduction in intracellular ROS

(DCFH-DA) and 4-HNE lipid peroxidation induced by ASP@PLGA-PEG in OGD/R-injured cells was markedly attenuated under ATF6 knockdown conditions (Fig. 14B), which was confirmed quantitatively by semi-quantitative fluorescence intensity analysis (Fig. 14C). Together, these results demonstrate that ATF6 acts upstream of NCOA4-mediated ferritinophagy and is essential for ASP@PLGA-PEG-mediated suppression of oxidative stress and ferroptosis.

### Biosafety assessment of ASP@PLGA-PEG

**Fig. 15:**
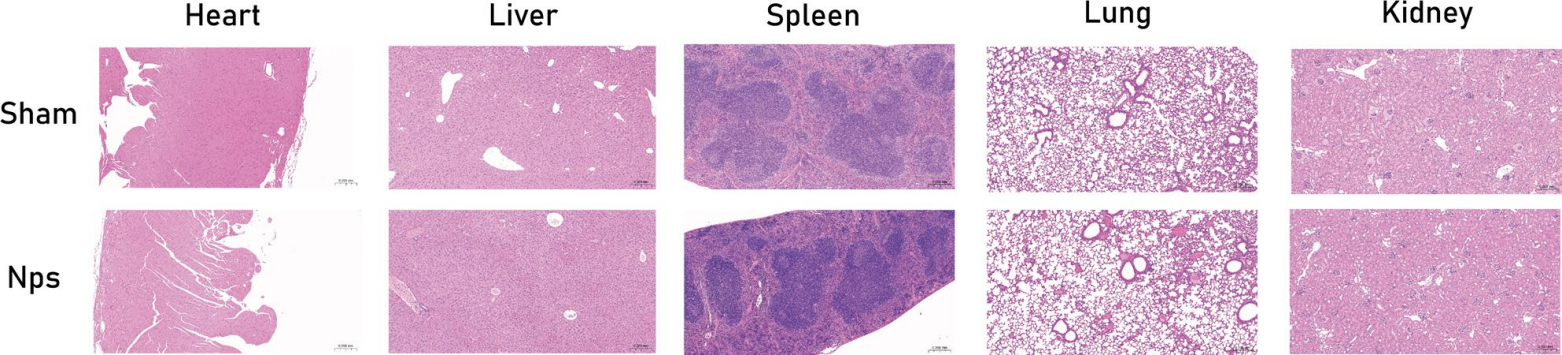
Histopathological evaluation of major organs following ASP@PLGA-PEG treatment. Representative H&E staining images of the heart, liver, spleen, lung, and kidney from Sham mice and mice treated with ASP@PLGA-PEG nanoparticles (nps). Scale bar = 200 μm

To further assess the systemic biosafety of ASP@PLGA-PEG nanoparticles, major organs were examined histologically (Fig. 15). H&E staining of the heart, liver, spleen, lung, and kidney revealed no evident pathological lesions, structural disruption, or inflammatory cell infiltration in the nanoparticle-treated mice compared with the Sham group. These findings indicate that ASP@PLGA-PEG nanoparticles do not induce detectable tissue toxicity under the experimental administration regimen.

## Discussion

In this study, we developed ASP@PLGA-PEG nanoparticles with a biphasic drug release profile. The drug adsorbed on the nanoparticle surface enables an initial rapid release, while a second, sustained release phase is provided by ASP encapsulated within the polymeric matrix. This dual-phase release protects the active ingredient and prolongs its therapeutic availability [29, 30]. In addition, PEG modification of PLGA allows the nanoparticles to evade clearance by the mononuclear phagocyte system (MPS), significantly extending their circulation time in vivo [31, 32]. We found no significant cytotoxicity in HL-1 cardiomyocytes exposed to ASP@PLGA-PEG for up to 48 hours, indicating their safety for injection. Compared with free ASP, the nanoparticles demonstrated higher bioavailability and lower toxicity due to their prolonged circulation and controlled release. Our previous studies confirmed the protective effects of ASP against myocardial MI/RI [17, 18], mainly due to its potent antioxidant properties. Here, we further validated this by testing ASP@PLGA-PEG in both an OGD/R cell injury model and a Langendorff isolated heart perfusion model. The results showed that ASP@PLGA-PEG significantly improved cardiomyocyte viability and reduced LDH release after OGD/R, outperforming free ASP. In the Langendorff model, ASP@PLGA-PEG treatment improved hemodynamic parameters and reduced infarct size, confirming that PLGA-PEG encapsulation enhances the protective effects of angelica polysaccharides.

ATF6 plays an essential role in MI/RI as a key regulator of the ERS response [33]. Hypoxia disrupts energy metabolism and protein folding in cardiomyocytes, leading to the accumulation of misfolded proteins. When reperfusion restores oxygen supply, excessive ROS production further damages the ER and mitochondria [34]. Under such stress, ATF6 is activated, translocated to the Golgi apparatus, cleaved by proteases, and releases its active fragment, which enters the nucleus to initiate transcription of genes that help restore ER function, such as GRP78/BiP. GRP78/BiP promotes correct protein folding and helps clear misfolded proteins, alleviating ER stress [35–38]. Persistent ER stress can activate CHOP, which triggers apoptosis. In this study, ASP@PLGA-PEG treatment increased the expression of ATF6 and BiP while decreasing CHOP levels, suggesting that the nanoparticles activate the ATF6 pathway to strengthen the unfolded protein response (UPR) and help maintain protein homeostasis under ischemic stress [33, 39, 40]. Notably, ASP@PLGA-PEG also reduced ROS levels, indicating that these nanoparticles not only alleviate ER stress but also mitigate oxidative damage, further supporting cardiomyocyte recovery after MI/RI. These findings support the idea that activating ATF6 and enhancing the UPR is a key mechanism by which ASP@PLGA-PEG protect the myocardium.

Ferroptosis, an iron-dependent form of programmed cell death, is characterized by lipid peroxidation triggered by iron accumulation [41]. ROS play a central role in ferroptosis by disturbing cellular redox balance and damaging membrane lipids, ultimately compromising membrane integrity [42, 43]. ER stress can amplify this process by increasing cellular sensitivity to ROS-induced lipid damage. Glutathione (GSH) is an essential antioxidant that counteracts ROS, and its oxidized form maintains the activity of GPX4, an enzyme that detoxifies lipid peroxides and protects against ferroptosis [44]. System xc^−^, a cystine/glutamate antiporter, supports GSH synthesis by importing cystine, the precursor for cysteine. When System xc^−^ function is impaired, GSH levels drop, oxidative stress increases, and ferroptosis is promoted [45]. In our study, ASP@PLGA-PEG regulated antioxidant enzyme activity, including catalase (CAT), GSH, and SOD, thereby enhancing the cell’s antioxidant capacity. This effect appears linked to ATF6 activation, although whether ATF6 directly modulates System xc^−^ remains to be clarified in future research.

Ferritin is the main intracellular iron storage protein that sequesters free iron to prevent oxidative damage [46]. Ferritin heavy chain 1 (FTH1) provides ferroxidase activity to convert ferrous iron (Fe^2 +^) into ferric iron (Fe^3 +^), safely storing it within the ferritin shell. During MI/RI, the protein NCOA4 promotes ferritin degradation by binding to ferritin and delivering it to autophagosomes for lysosomal degradation—a process known as ferritinophagy [47–49]. This process releases stored iron, raising free Fe^2 +^ levels in the cytoplasm, where it can drive the Fenton reaction to produce hydroxyl radicals that worsen oxidative damage [8, 50, 51]. Our results showed increased NCOA4 expression, elevated Fe^2 +^ levels, and enhanced LC3B-II conversion in the I/R group, indicating active ferritinophagy.

Transmission electron microscopy confirmed the presence of autophagosomes in ischemic myocardium.

ASP@PLGA-PEG treatment suppressed NCOA4 expression, reduced Fe^2 +^ accumulation, and lowered LC3B-II levels, suggesting inhibition of ferritinophagy. By blocking this pathway, ASP@PLGA-PEG limit free iron release and associated lipid peroxidation, ultimately protecting cardiomyocytes against ferroptosis during MI/RI.

Recent studies in different organs have consistently highlighted ferroptosis as a central driver of ischemia–reperfusion injury. In the liver, Qi et al. demonstrated that dimethyl fumarate alleviates hepatic I/R damage by activating the NRF2/SLC7A11/HO-1 axis, thereby restoring glutathione homeostasis and limiting lipid peroxidation [52]. Similarly, Zhang et al. reported that the BCAA metabolite L-β-aminoisobutyric acid (L-BAIBA) protects against lung I/R injury via the AMPK/Nrf2 pathway, which upregulates GPX4 and SLC7A11 and suppresses ferroptosis [53]. These findings, together with the transcriptomic analysis by Wei et al. identifying GPX4 and other ferroptosis-related genes as robust MIR signatures, support the concept that ferroptosis is a shared and druggable mechanism across multiple I/R-affected organs [54]. Our data extend this notion by demonstrating that, in the myocardium, ASP@PLGA-PEG not only reinforces the GPX4/SLC7A11 antioxidant axis but also attenuates ATF6-dependent ER stress and NCOA4-mediated ferritinophagy, thereby reducing Fe^2 +^ overload and iron-driven lipid peroxidation.

Beyond ferroptosis, necroptosis has been recognized as another major form of regulated cell death contributing to MI/R, often interacting with autophagy, mitochondrial damage, and ferroptosis [55].

Although we did not directly assess RIPK1/RIPK3/MLKL signaling, the robust inhibition of ROS generation and preservation of mitochondrial integrity by ASP@PLGA-PEG suggest that this nanoplatform may also modulate additional death pathways, which warrants further investigation. Moreover, the observation by Jankauskas et al. that serum from non-surviving COVID-19 patients induces a ferroptotic signature in human endothelial cells—characterized by decreased GPX4, SLC7A11, and FTH1 and increased lipid peroxidation—is highly consistent with the molecular pattern we observed in cardiomyocytes during MI/R [56]. Together, these studies underscore the broader relevance of ferroptosis and iron dysregulation in cardiovascular and systemic vascular injury and suggest that targeting ER stress–driven ferritinophagy, as achieved by ASP@PLGA-PEG, may represent a generally applicable strategy to limit iron-dependent cell death.

Taken together, our findings indicate that ASP@PLGA-PEG nanoparticles exert cardioprotective effects through a multi-faceted mechanism involving ATF6-mediated alleviation of endoplasmic reticulum stress, enhancement of the cellular antioxidant system, and suppression of NCOA4-mediated ferritinophagy, which collectively help maintain mitochondrial function and reduce iron-dependent lipid peroxidation during ischemia-reperfusion injury.

From a translational perspective, ASP@PLGA-PEG has several potential advantages as a therapeutic candidate for MI/R. ASP is derived from a traditional Chinese medicinal herb with a favorable historical safety profile, whereas PLGA-PEG is a biocompatible and biodegradable polymer that has already been used in clinically approved drug delivery systems. The prolonged circulation, controlled release behavior and enhanced cardioprotection observed in both ex vivo and in vivo models suggest that ASP@PLGA-PEG may be suitable for peri-procedural administration during reperfusion therapies such as PCI. Nonetheless, before clinical application, further studies are required to characterize its pharmacokinetics, optimal dosing, immunogenicity and long-term safety in large-animal models.

However, this study has some limitations. Our in vitro siATF6 experiments showed that knockdown of ATF6 almost completely abolished the regulatory effects of ASP@PLGA-PEG on NCOA4 and FTH1, and attenuated its antioxidative and anti-ferroptotic actions, suggesting that ATF6 is functionally upstream of NCOA4-mediated ferritinophagy and ferroptosis. However, the precise molecular mechanisms by which ATF6 signaling influences ferritinophagy—such as whether it directly regulates the transcription of NCOA4, FTH1, LC3B and related targets, or acts indirectly through intermediate pathways—remain incompletely understood and warrant further investigation. Future studies should also systematically validate the presence and temporal characteristics of the ATF6–NCOA4–ferritinophagy axis in intact myocardial tissue and large-animal models, and elucidate its interactions with iron-metabolism regulatory networks under MI/R conditions. Clarifying these issues will help to further refine and substantiate the therapeutic potential of ASP@PLGA-PEG as an intervention for myocardial ischemia–reperfusion injury.

## Conclusion

In conclusion, this study successfully developed ASP@PLGA-PEG nanoparticles and demonstrated their potent antioxidant and cardioprotective effects in vitro, ex vivo, and in vivo. We found that ASP@PLGA-PEG nanoparticles activate ATF6 to alleviate endoplasmic reticulum stress in myocardial ischemia–reperfusion injury, thereby reducing oxidative stress and lipid peroxidation. This cascade effect suppresses NCOA4-mediated ferritinophagy and ultimately prevents iron-dependent ferroptosis. These findings reveal new biological functions of ASP delivered via a PLGA-PEG nanocarrier and clarify the key mechanisms underlying its protective action against MI/R. We propose that targeting excessive ferritinophagy represents a promising therapeutic strategy for MI/R, and that ASP@PLGA-PEG nanoparticles hold substantial potential for further preclinical investigation and future translational application in myocardial infarction therapy.

## Electronic supplementary material

Below is the link to the electronic supplementary material.

Supplementary Material 1

Supplementary Material 2

## Data Availability

Data for this paper can be provided by the corresponding author upon request.
