# Supplementary material for: Angelica sinensis polysaccharide nanoparticles can improve myocardial ischemia-reperfusion injury by inhibiting ferritinophagy via the ATF6/NCOA4 pathway
Source: J Transl Med. 2026 Feb 26;24:460. doi: 10.1186/s12967-026-07752-8 (PMC13040952; doi:10.1186/s12967-026-07752-8)
Supplement: Supplementary file 1 — Supplementary Material 1 [file 12967_2026_7752_MOESM1_ESM.doc]

Fig.S1

| Antibody | company | Catalog number |
| --- | --- | --- |
| Anti-Glutathione Peroxidase 4 Rabbit Monoclonal Antibody | Abcam | ab125066 |
| xCT Rabbit mAb | Abmart | T57046 |
| 4-Hydroxynonenal Mouse Monoclonal Antibody | Thermofisher | MA5-27570 |
| ATF6 Polyclonal antibody | Proteintech | 24169-1-AP |
| BiP (C50B12) Rabbit mAb | Cell Signaling Technology | 3177T |
| Anti-DDIT3(CHOP) Mouse Monoclonal Antibody | Abcam | ab11419 |
| Anti-NCOA4 Rabbit Monoclonal Antibody | Abcam | ab314553 |
| Anti-Ferritin Heavy Chain Rabbit Monoclonal Antibody | Abcam | ab183781 |
| Anti-LC3B Rabbit Monoclonal Antibody | Abcam | Ab192890 |
| Beta Actin Monoclonal antibody | Proteintech | 66009-1-Ig |
| GAPDH Monoclonal antibody | Proteintech | 60004-1-Ig |

Table S1 Information of antibodies used in this study.
