# Supplementary figures and images for: Angelica sinensis polysaccharide nanoparticles can improve myocardial ischemia-reperfusion injury by inhibiting ferritinophagy via the ATF6/NCOA4 pathway
Source: J Transl Med. 2026 Feb 26;24:460. doi: 10.1186/s12967-026-07752-8 (PMC13040952; doi:10.1186/s12967-026-07752-8)

**Fig-7**

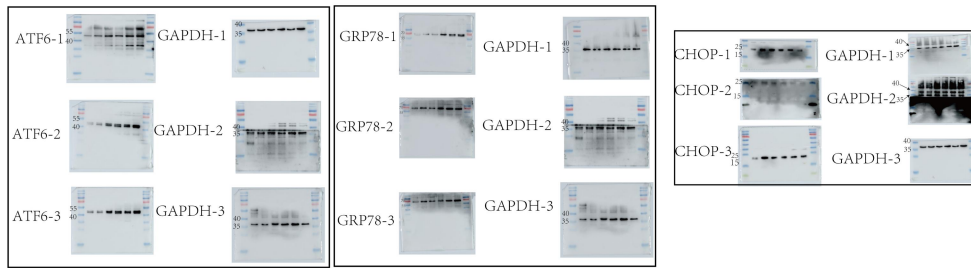

**Fig-8**

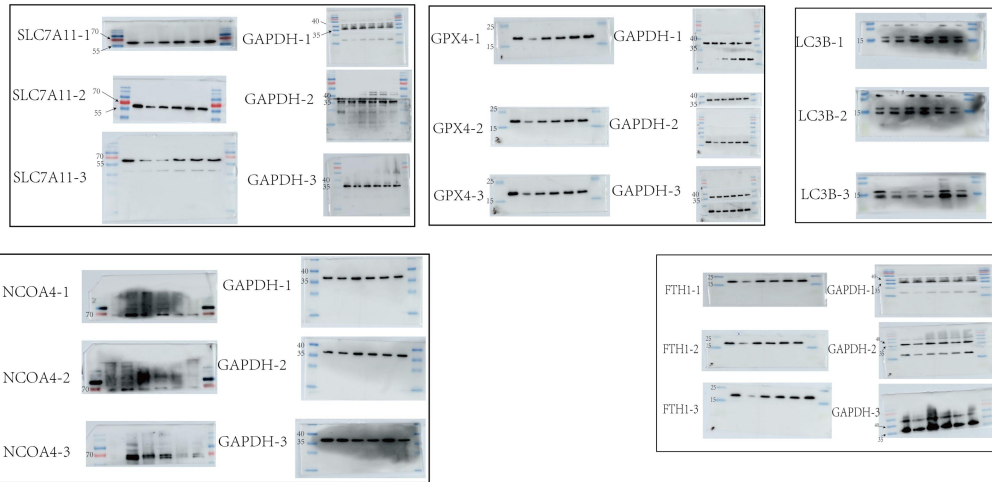

**Fig-10**

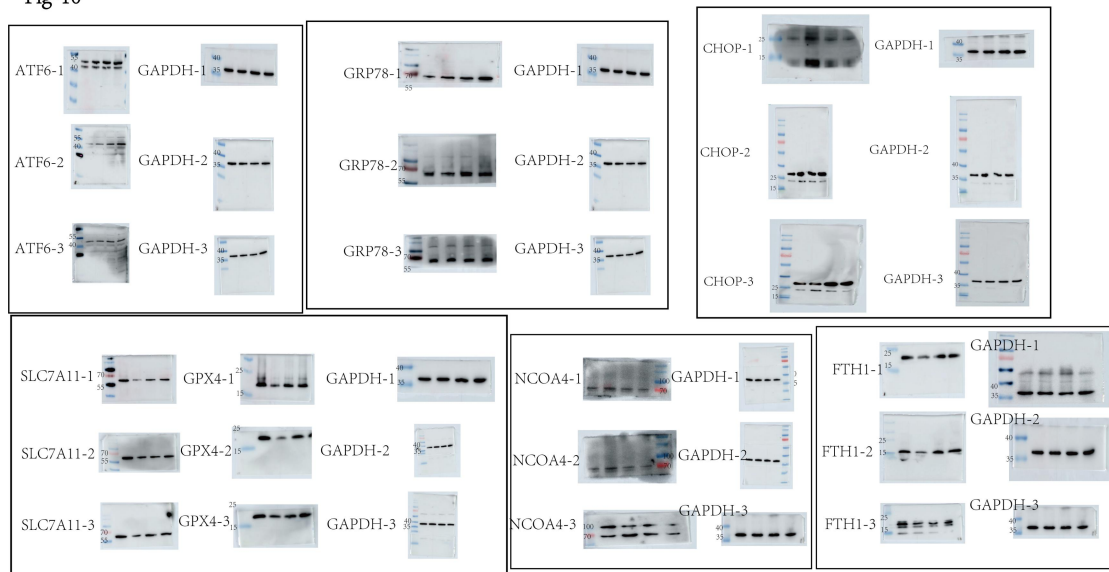

Fig-11

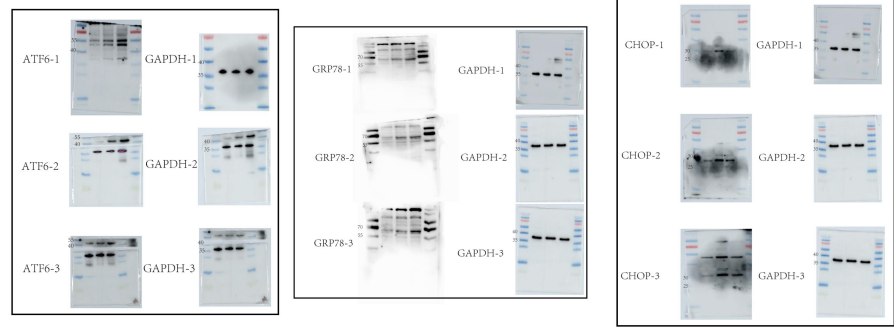

Fig-13

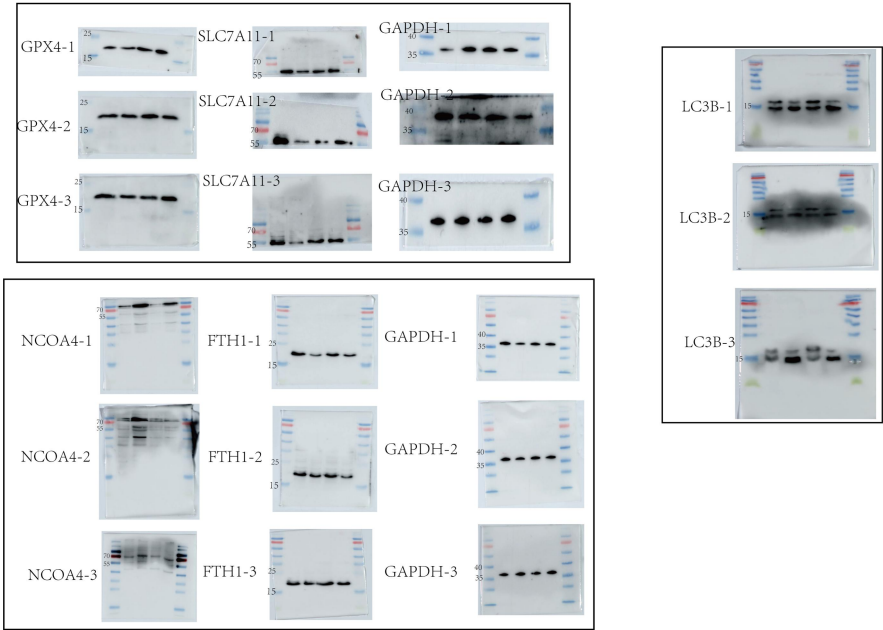

Fig-14

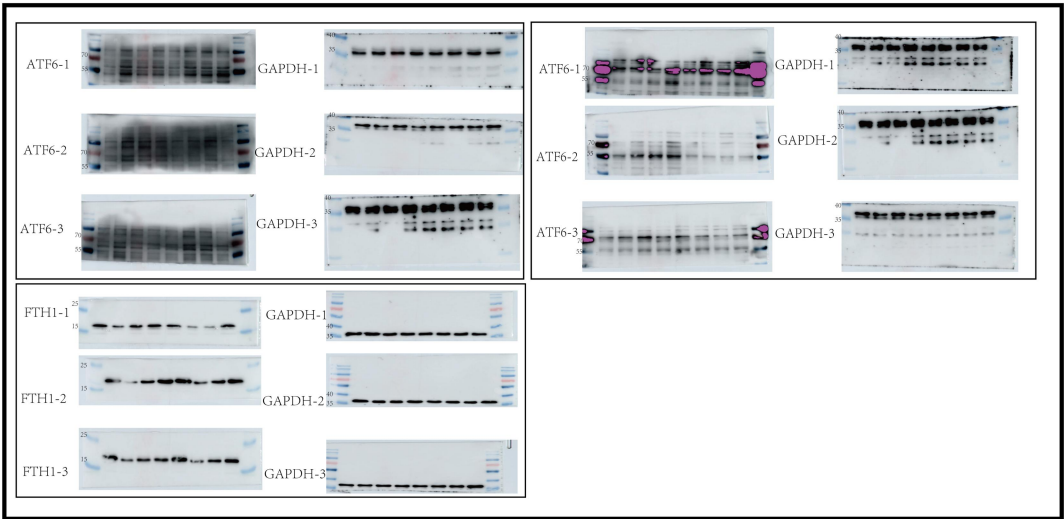

Supplement: Supplementary file 2 — Supplementary Material 2 [file 12967_2026_7752_MOESM2_ESM.pdf]
